# CCN2 deficiency in smooth muscle cells triggers cell reprogramming and aggravates aneurysm development

**DOI:** 10.1172/jci.insight.162987

**Published:** 2023-01-10

**Authors:** Yu Wang, Xuesong Liu, Qian Xu, Wei Xu, Xianming Zhou, Andrew Leask, Zhiyong Lin

**Affiliations:** 1 Cardiology Division, Emory University School of Medicine, Atlanta, Georgia, USA.; 2 Department of Cardiology, The First Affiliated Hospital of Kunming Medical University, Kunming, China.; 3 Department of Cardiovascular Surgery, Union Hospital, Tongji Medical College, Huazhong University of Science and Technology, Wuhan, China.; 4 University of Saskatchewan, College of Dentistry, Saskatoon, Saskatchewan, Canada.

**Keywords:** Vascular Biology, Extracellular matrix

## Abstract

Vascular smooth muscle cell (SMC) phenotypic switching is widely recognized as a key mechanism responsible for the pathogenesis of several aortic diseases, such as aortic aneurysm. Cellular communication network factor 2 (CCN2), often upregulated in human pathologies and animal disease models, exerts myriad context-dependent biological functions. However, current understanding of the role of SMC-CCN2 in SMC phenotypic switching and its function in the pathology of abdominal aortic aneurysm (AAA) is lacking. Here, we show that SMC-restricted CCN2 deficiency causes AAA in the infrarenal aorta of angiotensin II–infused (Ang II–infused) hypercholesterolemic mice at a similar anatomic location to human AAA. Notably, the resistance of naive C57BL/6 WT mice to Ang II–induced AAA formation is lost upon silencing of CCN2 in SMC. Furthermore, the pro-AAA phenotype of SMC-CCN2-KO mice is recapitulated in a different model that involves the application of elastase–β-aminopropionitrile. Mechanistically, our findings reveal that CCN2 intersects with TGF-β signaling and regulates SMC marker expression. Deficiency of CCN2 triggers SMC reprograming associated with alterations in Krüppel-like factor 4 and contractile marker expression, and this reprograming likely contributes to the development of AAA in mice. These results identify SMC-CCN2 as potentially a novel regulator of SMC phenotypic switching and AA biology.

## Graphical abstract

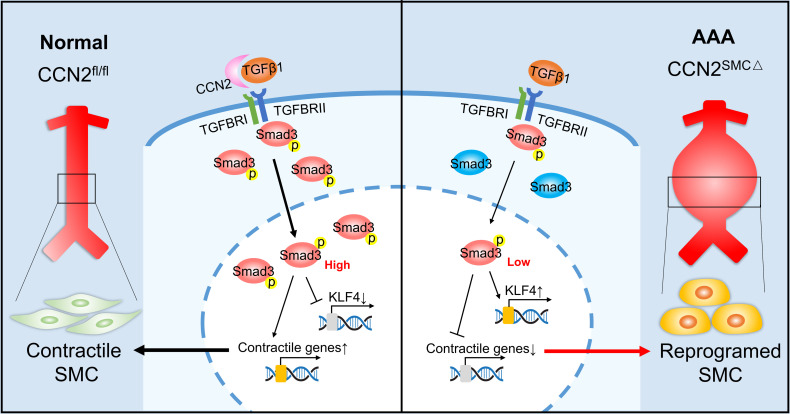

## Introduction

Aortic aneurysm is a life-threatening vascular pathology that poses significant challenges to health care systems worldwide. Despite the advances in medical science and technology, surgical or endovascular interventions remain the only available therapies for high-risk patients. Findings from human and animal studies have suggested common aneurysm pathophysiology involving vascular endothelial and smooth muscle cell (SMC) dysfunction, extracellular matrix (ECM) deterioration, and increased vascular inflammation (1, 2). However, a comprehensive understanding of the aneurysm biology has yet to be acquired. Sticking points include lack of early-stage tissue samples and the presence of confounding factors, such as atherosclerosis and hypertension, that can be major obstacles to the next therapeutic leap. In addition, even with the help of animal models, very few studies were carefully designed to dissect the early molecular triggers in a cell-specific manner, which the multifactorial, multistage nature of aneurysm development is likely to rely upon.

Cellular communication network factor 2 (CCN2), also known as connective tissue growth factor (CTGF), is a member of the CCN matricellular protein family. Similar to the majority of the other CCN proteins, CCN2 plays critical roles in the regulation of normal cell function and signaling. CCN2 conveys its biological effects through interaction with specific receptors (e.g., integrins, LRP1, HSPG) (3), the ECM protein fibronectin (4), and cytokines such as TGF-β and VEGF (5, 6). Although initially best known for promoting tissue fibrosis in multiple fibrotic diseases, CCN2 has also been implicated in cancer (7), inflammatory diseases (8), neural (9) and ocular disorders (10), and autoimmune diseases (11, 12). In vascular pathologies, increase of CCN2 has been reported in atherosclerosis (13, 14), aortic aneurysms (15), aorta dissection (16), and restenosis (17). Published studies hint at a causal role of elevated CCN2 in these diseases, largely owing to CCN2’s established function in promoting the formation and remodeling of the ECM (18, 19). However, in part due to the lack of critical genetic evidence derived from transgenic animals, the precise physiological role of CCN2 from different cellular origins in vivo remains largely undefined for vascular diseases including aortic aneurysm.

SMC integrity and function are central for maintaining vascular homeostasis. Alterations in SMC function, as well as transdifferentiation of SMC populations, have long been hypothesized to play important roles in aneurysm formation, with evidence documented in both abdominal aortic aneurysm (AAA) and thoracic aortic aneurysm (TAA). In patients with Marfan or Loeys-Dietz syndrome, aneurysm development has been associated with an activated TGF-β signaling in SMCs (20). However, a recent study using SMC-specific TGFBR2-deficient mice demonstrated that a loss of smooth muscle TGF-β signaling, when combined with hyperlipidemia, stimulates the transdifferentiation of media SMC to a mesenchymal stem cell–like (MSC-like) state and promotes aortic aneurysm (21). Given that (a) CCN2 is a potential downstream target of TGF-β signaling (22) and (b) CCN2 has been suggested to potentiate TGF-β signaling, including TGF-β’s effect on inducing SMC contractile marks (3), it is conceivable to speculate that SMC-specific CCN2 may be critical for maintaining vascular homeostasis.

The present study uses tissue-specific inducible CCN2-deficient mice (CCN2^^fl/fl^^–Myh11–Cre-ER^^T2^^) and murine models of AAA (induced by systemic angiotensin II [Ang II] infusion or local adventitia elastase exposure) to characterize the role of SMC-specific CCN2 in the development of AAA. We show that ablation of CCN2 in SMCs results in an unanticipated AAA phenotype in the infrarenal aortic region, a location dissimilar to Ang II–induced murine AAA yet identical to human AAA pathology. Subsequent in vivo and cell culture–based studies point to SMC phenotypic switching as a key driver predisposing the AAA phenotype in SMC-CCN2–deficient mice. Our studies provide evidence supporting the protective role of SMC-CCN2 against AAA.

## Results

### CCN2 protein is upregulated in both human and mouse AAA tissues.

**Figure 1:**
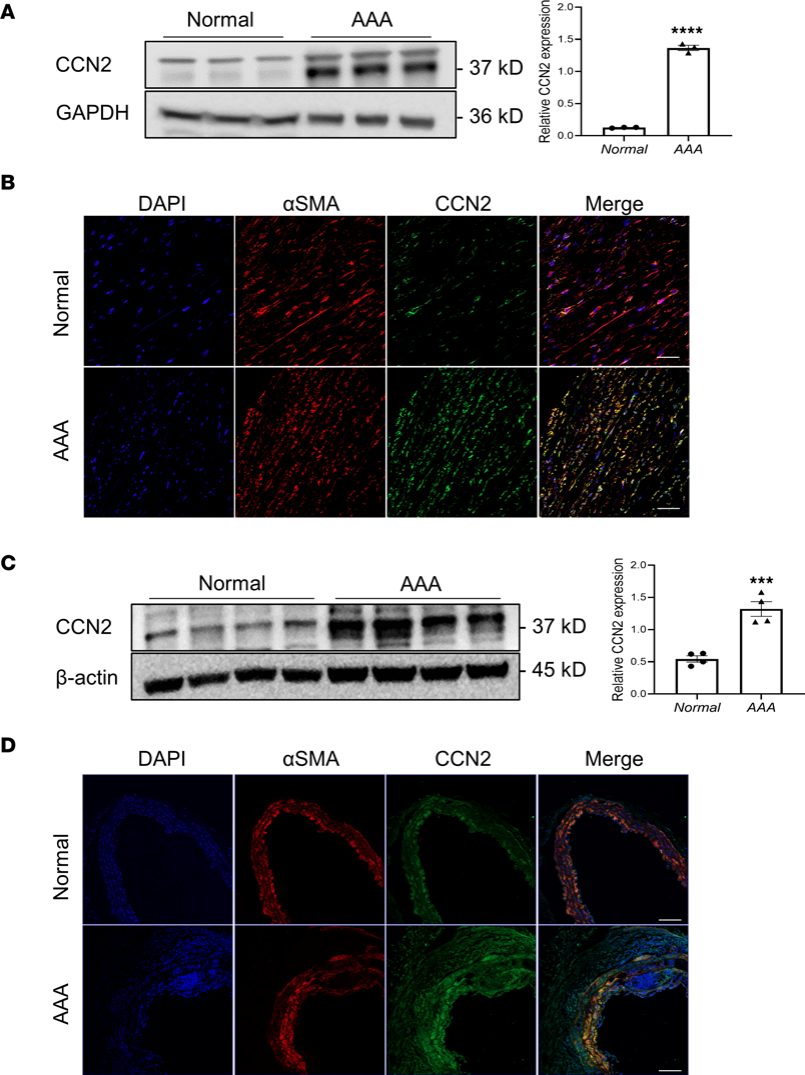
CCN2 protein is upregulated in both human and mouse abdominal aortic aneurysm (AAA) tissues. (**A**) Western blot analysis of CCN2 protein in healthy human aortas versus AAA samples (*n* = 3). (**B**) Representative immunofluorescence staining images of CCN2 in human aortic sections with or without AAA (*n* = 3). (**C**) Western blot analysis of mouse CCN2 protein in abdominal aortic tissue lysates from normal (saline-treated) versus AAA (Ang II–treated) samples (*n* = 4). (**D**) Representative immunofluorescence staining images of CCN2 in mouse aortic sections with or without AAA (*n* = 3). Scale bars: 100 μm. Data were quantified and represented as mean ± SEM. ***P < 0.001, ****P < 0.0001, 2-tailed Student’s *t* test.

Little information is available regarding the impact of CCN2 modulation in vascular diseases. Studies performed to date are inconclusive, as the levels of CCN2 expression reported in human aneurysms varies and is, at times, contradictory (16, 21, 23). To clarify this, we compared the CCN2 protein levels between normal human aortic tissue and AAA samples. CCN2 protein was expressed minimally in normal aortas yet was remarkably induced in aortas with aneurysms (Figure 1A). Immunofluorescence staining of the aortic tissue revealed that increased CCN2 expression was mostly observed in the media layer and was colocalized with SMC marker αSMA (Figure 1B), suggesting that SMC is an important source of vascular CCN2 in response to vascular injury. A similar increase of CCN2 protein was observed in Ang II–induced AAA samples compared with aortas from saline-treated mice (Figure 1, C and D). It is unclear, however, whether a boost in CCN2 levels in the vasculature is a reparative mechanism to combat aneurysm progression, an agitator, or simply a consequence with little relevance of disease pathology. Since vascular SMCs (VSMCs) contribute to a large percentage of total CCN2 in the vascular bed and considering that SMC dysfunction has long been implicated in the development of AAA (24, 25), we explored the impact of SMC-specific CCN2 deficiency in the development of AAA in mice.

### Deficiency of CCN2 in SMC exacerbates Ang II–induced AAA in hypercholesterolemia mice.

**Figure 2:**
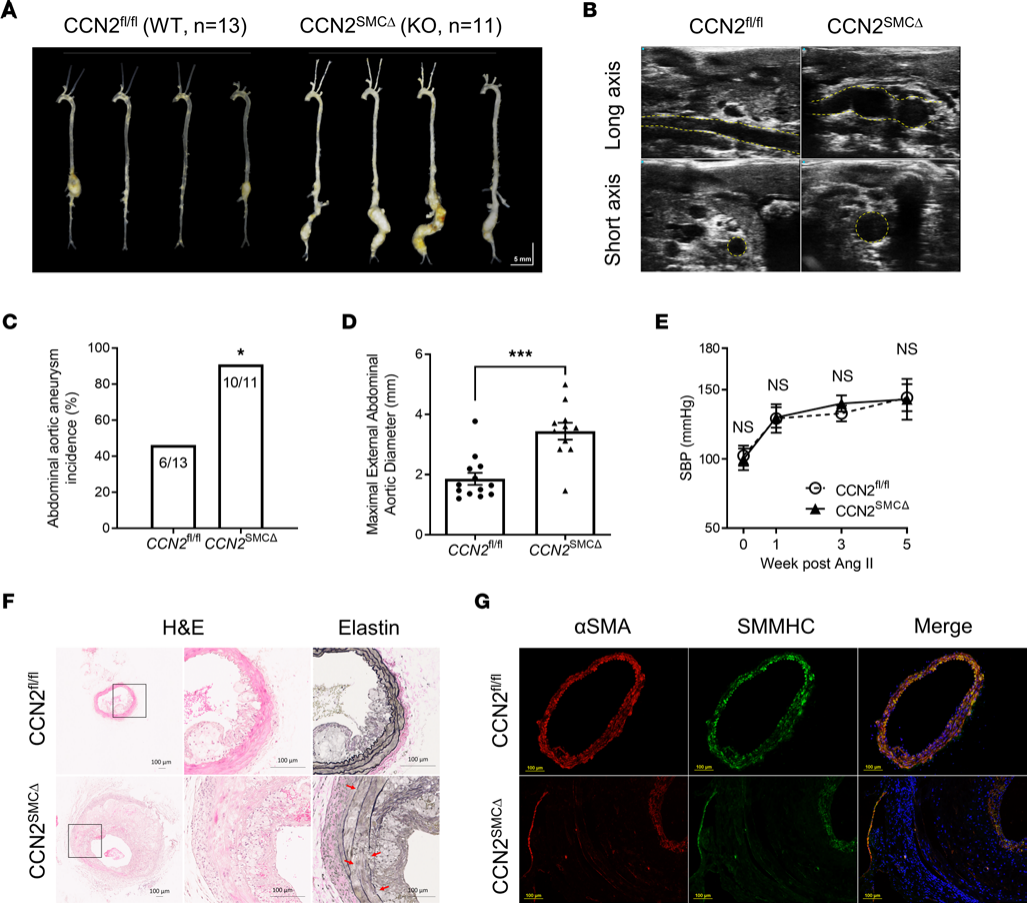
SMC-specific deficiency of CCN2 exacerbates Ang II–induced AAA in hypercholesterolemia mice. (**A**) Representative gross images of aortas from CCN2^fl/fl^ (WT, *n* = 13) and CCN2^SMCΔ^ (KO, *n* = 11) mice infused with Ang II. (**B**) Representative abdominal ultrasound images (long- and short-axis views) of mice in both groups. (**C**) AAA incidence in mice from both groups. *P < 0.05, χ^2^ test. (**D**) Maximal external aortic diameters at study endpoint in mice from both groups. Data were quantified and represented as mean ± SEM. ***P < 0.001, 2-tailed Student’s *t* test. (**E**) Systolic blood pressure (SBP) measured at various time points before and after Ang II infusion for mice in both groups (*n* = 8). (**F**) Representative images of H&E-stained (5× and 20×) and Elastin-stained (20×) abdominal aortic sections from both groups (*n* = 5). Red arrows indicate elastin break points. (**G**) Immunofluorescence double staining of abdominal aortic sections from both groups with specific antibodies against αSMA (red) and SMMHC (green). Nuclei were stained with DAPI (blue). *n* = 4. Scale bars: 100 μm.

To evaluate the impact of SMC-CCN2 deficiency on murine AAA, we first generated CCN2^^fl/fl^^–Myh11–Cre-ER^^T2^^ mice by crossing CCN2-floxed mice with Myh11–Cre-ER^^T2^^. At 8 weeks of age, maleCCN2^^fl/fl^^–Myh11–Cre-ER^^T2^^ mice were subjected to 5 consecutive days of tamoxifen injection to induce CCN2 deletion in SMCs. After an additional 2 weeks, the resultant mice and CCN2-floxed control animals were rendered hypercholesterolemic (achieved via AAV-proprotein convertase subtilisin/kexin type 9 [PCSK9] injection to deplete LDL receptor [LDLR]) followed by Ang II infusion. Systemic Ang II infusion at the dose of 500 ng/kg/min successfully induced AAA in hypercholesterolemic mice in both groups. In the CCN2^^fl/fl^^ group, 46% of the mice developed aneurysms in the suprarenal region of the abdominal aortas, while 91% of the SMC-specific CCN2-KO mice, henceforth CCN2^^SMCΔ^^ mice, developed aneurysms exclusively in the infrarenal segments, the identical anatomic location where human AAA occurs (Figure 2, A and C). Gross and ultrasound data show that the aneurysms in CCN2^^SMCΔ^^ groups exhibited greater uniformity and size (Figure 2, B and D) and were particularly extensive (from renal branch to femoral bifurcation) and tortuous, reminiscent of advanced human aortic aneurysms. No differences in systolic blood pressure (SBP) were observed between groups at various time points before and after Ang II infusion (Figure 2E). Histological analysis of the aneurysm sections of the CCN2^^SMCΔ^^ mice revealed expected changes, including severe media deterioration, elastin degradation, and immune cell infiltration (Figure 2F and Supplemental Figure 1, A and B; supplemental material available online with this article; https://doi.org/10.1172/jci.insight.162987DS1). Immunofluorescence studies confirmed massive aortic wall dilation and media thickening; moreover, cells in the expanded media layer and adventitia showed minimum expression of SMC contractile markers, αSMA and smooth muscle myosin heavy chain (SMMHC) (Figure 2G). Notably, in CCN2^^SMCΔ^^ mice, diminished expression of SMC markers was also evident in regions of the aorta with no aneurysm or dilations (Supplemental Figure 1C), suggesting intrinsic molecular changes associated with SMC CCN2 deficiency prior to the development of aneurysm.

### Ang II alone induces infrarenal aortic aneurysm formation in CCN2^SMCΔ^ mice.

**Figure 3:**
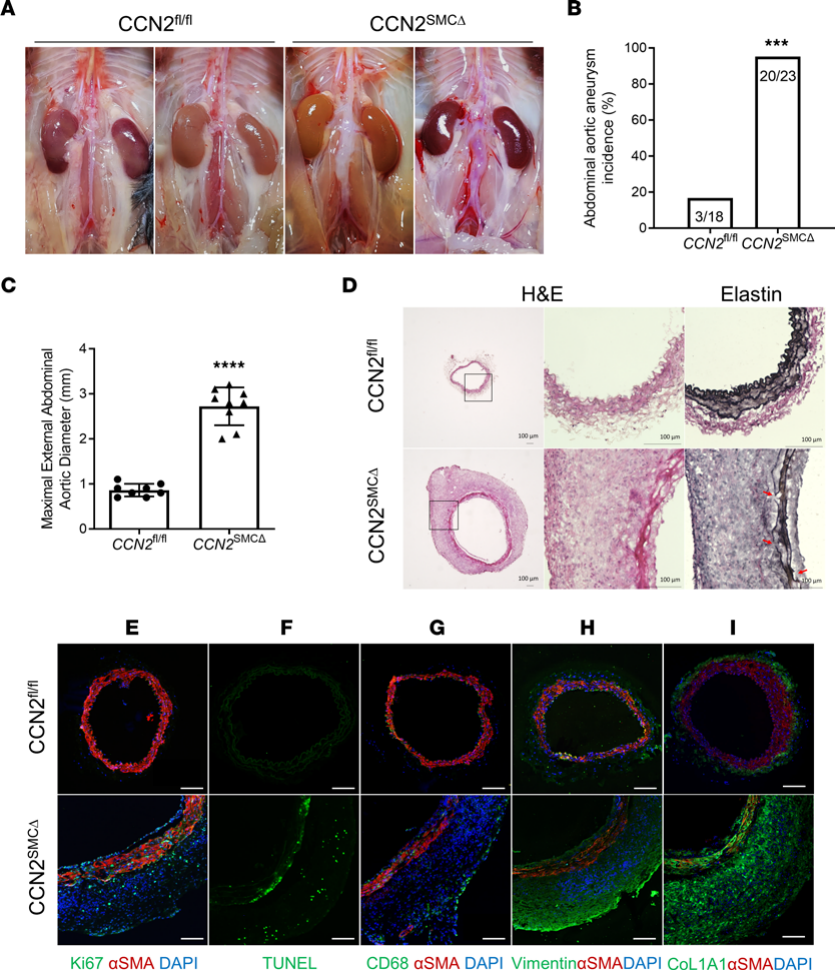
SMC-specific deficiency of CCN2 results in the development of infrarenal AAA in mice with 7-day Ang II infusion. (**A**) Representative gross images of abdominal aortas from CCN2^fl/fl^ (*n* = 18) and CCN2^SMCΔ^ (*n* = 21) mice with Ang II infusion. (**B**) AAA incidence in mice from both groups. ***P < 0.001, χ^2^ test. (**C**) Maximal external aortic diameters at study endpoint (*n* = 9). Data were quantified and represented as mean ± SEM. ****P < 0.0001, 2-tailed Student’s *t* test. (**D**) Representative images of H&E-stained (5× and 20×) and Elastin-stained (20×) infrarenal aortic sections from both groups. Red arrows indicate elastin break points. *n* = 5. (**E** and **G**–**I**) Immunofluorescence double staining of αSMA (red) and Ki67 (green) (**E**), αSMA (red) and CD68 (green) (**G**), αSMA (red) and Vimentin (green) (**H**), and αSMA (red) and collagen I (green) (**I**) in infrarenal aortic sections from Ang II–infused CCN2^fl/fl^ and CCN2^SMCΔ^ mice. *n* = 4. Nuclei were stained with DAPI (blue). (**F**) In situ cell death assay of infrarenal aortic sections from CCN2^fl/fl^ and CCN2^SMCΔ^ mice; positive green signals indicate apoptotic cells. *n* = 4. Scale bars: 100 μm.

The profound AAA pathology manifestations in hypercholesterolemic CCN2^^SMCΔ^^ mice prompted us to speculate on what might occur to these animals when infusing Ang II without being hypercholesterolemic. With this in mind, and to capture early cellular changes and explore possible molecular mechanisms fueling aneurysm development in CCN2^^SMCΔ^^ mice, we infused Ang II (500 ng/kg/min) or saline (as vehicle control) to CCN2^^fl/fl^^ and CCN2^^SMCΔ^^ mice. At day 7, mice were euthanized for tissue harvesting. To our surprise, Ang II alone was sufficient to induce remarkable AAA development in 20 of 21 mice examined in the CCN2^^SMCΔ^^ group (again in the infrarenal aortic region), with most CCN2^^SMCΔ^^ mice exhibiting strong “ballooning” of abdominal and elongated aorta. In contrast, only a few (3 of 18 mice) developed mild aneurysms in the thoracic or suprarenal region of the aorta in the control group (Figure 3, A–C). Consistent with higher AAA incidence (Figure 3B), in CCN2^^SMCΔ^^ mice, the maximum abdominal aortic diameters of aneurysms in the CCN2^^SMCΔ^^ group were significantly larger than those in the control group (Figure 3C; 0.86 mm versus 2.72 mm, *P* < 0.0001).

Similar to what is observed in the long-term Ang II model (Figure 1), massive wall expansion, media thickening, and elastin breakdown were evident but with no sign of lipid accumulation or atherosclerosis in the aneurysm sites from the CCN2^^SMCΔ^^ group (Figure 3D and Supplemental Figure 2A). Cells in the thickened media layer exhibited increased proliferation, as indicated by Ki67^^+^^ staining (Figure 3E) and apoptosis (Figure 3F), suggesting accelerated cell turnover. Moreover, most cells in the remodeled area were CD68^^–^^ and showed little to no expression of αSMA (Figure 3G), but they exhibited strong vimentin and collagen I staining, markers for mesenchymal-like cells (Figure 3, H and I), suggesting a distinctive cell population from conventional SMCs. Correspondingly, tissue MMP activity was also elevated in the aneurysm sites (Supplemental Figure 2B). However, this is likely not attributed to the infiltration of immune cells, since only scarce and sporadic CD68^^+^^ cells were observed mostly along the edge of the adventitia (Figure 3G). Expectedly, all of the cellular and molecular changes stated above were not seen in the saline-treated groups (Supplemental Figure 3). Considering the important role of SMC apoptosis in aneurysm pathology, we also studied the effects of CCN2 deficiency on apoptosis in cultured human primary aortic SMCs (HASMCs). In line with augmented aortic cell apoptosis in Ang II–treated CCN2^^SMCΔ^^ mice (Figure 3F), deficiency of CCN2 resulted in a significant increase in Staurosporine-induced SMC apoptosis (Supplemental Figure 4A) and elevated caspase 8 and caspase 3 activities (Supplemental Figure 4B), supporting CCN2 as an important factor for SMC survival against harmful insults.

### Exacerbation of elastase-BAPN induced infrarenal aortic aneurysm in CCN2^SMCΔ^ *mice*.

To corroborate that the aneurysm phenotype was not limited to the Ang II model, we tested the consequence of SMC-specific CCN2 deficiency in the elastase–β-aminopropionitrile (BAPN) model (26). A brief 10-minute soaking of abdominal mouse aortas with topical elastase in conjunction with 14 days of BAPN supplementation in the drinking water successfully induced AAA formation in both CCN2^^fl/fl^^ and CCN2^^SMCΔ^^ mice. However, the sizes of the dilations were markedly bigger in CCN2^^SMCΔ^^ mice compared with controls, as can be seen through gross images (Supplemental Figure 5A) and the ultrasonic measurements of maximal external diameters of the abdominal aortas (Supplemental Figure 5, B and C). In addition, greater aortic wall expansion was observed in KO mice, with the remodeling and thickening of the media layers more severe in KO mice but to a lesser extent when compared with those in the Ang II model (Supplemental Figure 5D). Notably, the losses of media elastin were comparable between the 2 groups, with a complete loss at the anterior and residual elastin at the posterior of the aortic wall, suggesting that the differences in aneurysms were not the consequence of the surgical variation (Supplemental Figure 5D). Since the Ang II model is arguably more clinically relevant, and considering the possible interplay between CCN2 and Ang II (27), we decided to use the Ang II model for subsequent studies.

### RNA-Seq analysis reveals SMC-derived CCN2 participates in vascular smooth muscle contraction and transmembrane receptor signaling.

**Figure 4:**
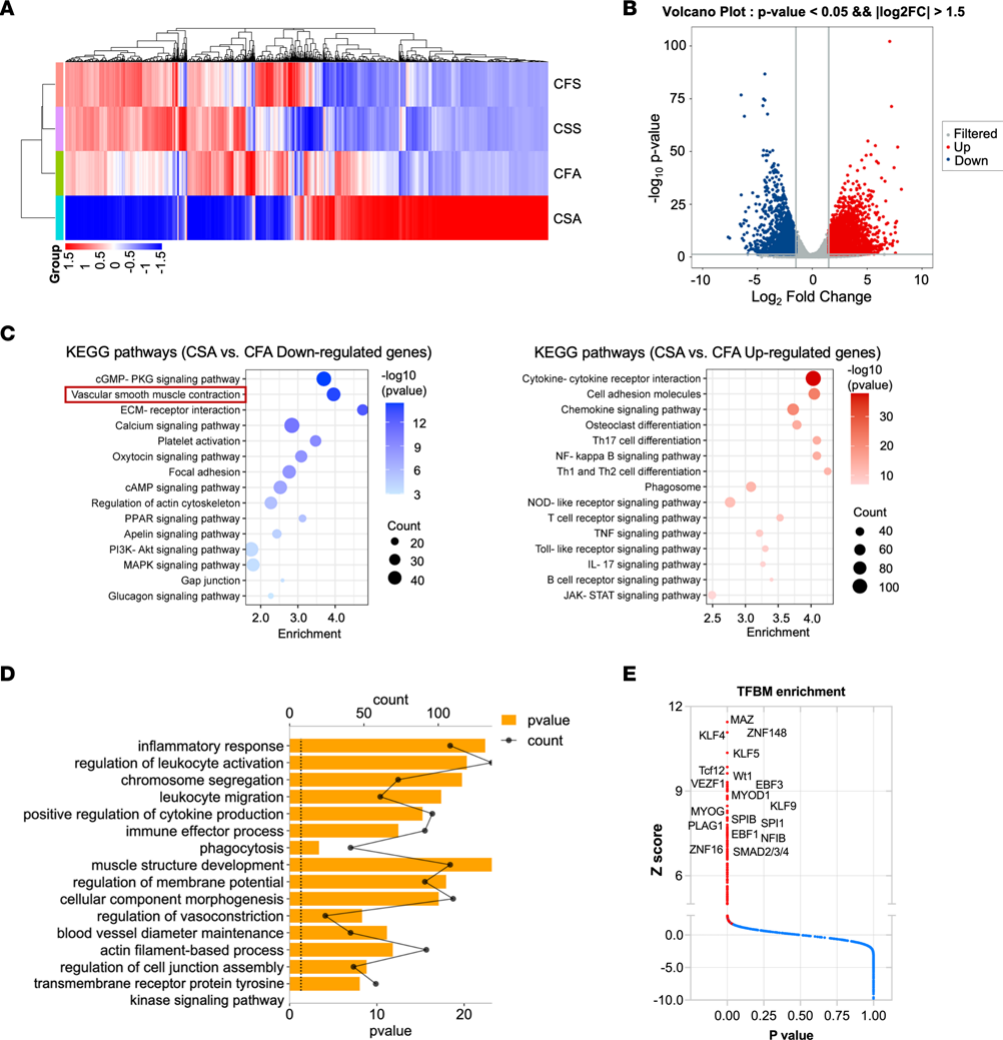
RNA-Seq analysis reveals SMC-derived CCN2 participates in vascular smooth muscle contraction and transmembrane receptor signaling. (**A**) Heatmap of differentially expressed genes (DEGs) in the CFS (CCN2^fl/fl^ + Saline), CSS (CCN2^SMCΔ^ + Saline), CFA (CCN2^fl/fl^ + Ang II), and CSA (CCN2^SMCΔ^ + Ang II) aortic tissues (*n* = 3), as obtained by RNA-Seq (FDR < 0.05). (**B**) A volcano plot showing DEG expression between CFA and CSA aortic tissues. Genes in colored dots have an adjusted *P* < 0.05 and |log_2_ fold change| > 1.5. (**C**) Top enriched KEGG pathways analysis of RNA-Seq data obtained as in **B**. (**D**) Top enriched GO pathways analysis of RNA-Seq data obtained as in **B**. (**E**) Transcription factor binding motif (TFBM) enrichment analysis by pScan of RNA-Seq data obtained as in **B**. Colored dots indicate significant hits. *P* < 0.05 in a *z* test.

To unbiasedly evaluate the functional role of SMC-derived CCN2 in AAA, we isolated total RNA from infrarenal abdominal aortic tissue samples in CCN2^^fl/fl^^ and CCN2^^SMCΔ^^ mice infused with saline or Ang II for RNA-Seq. The data revealed drastically different gene expression profiles associated with CCN2 deficiency in SMC even under saline treatment. Much greater divergences in gene expression were discovered between groups under Ang II treatment (Figure 4A), in which 3,723 genes were upregulated and 2,413 genes were downregulated in aortic tissues of CCN2^^SMCΔ^^ mice compared with those of control mice (Figure 4B). We then performed KEGG and GO pathway analysis on differentially expressed genes to explore biological processes important for AAA development in mice with SMC-specific deficiency. Data suggest that the top affected processes include ECM-receptor interactions, vascular smooth muscle contraction (Figure 4C), inflammation, and transmembrane receptor protein tyrosine kinase signaling (Figure 4D). To explore why deficiency of CCN2, an ECM signaling protein, could lead to enormous differences in gene expression, transcription factor binding motif (TFBM) enrichment analysis was performed. Expectedly, transcription factors with well-documented roles in SMC biology and aneurysm, including Krüppel-like factors (KLFs; KLF4, KLF5) (28–30) and Smads (Smad2, Smad3, Smad4) (31–33), were significantly affected in response to SMC-specific CCN2 deficiency (Figure 4E). These findings provided additional guidance for the subsequent exploration of the molecular traits of AAA formation in SMC-specific CCN2-deficient mice.

### SMC-specific deficiency of CCN2 represses SMC contractile markers both in vivo and in vitro.

**Figure 5:**
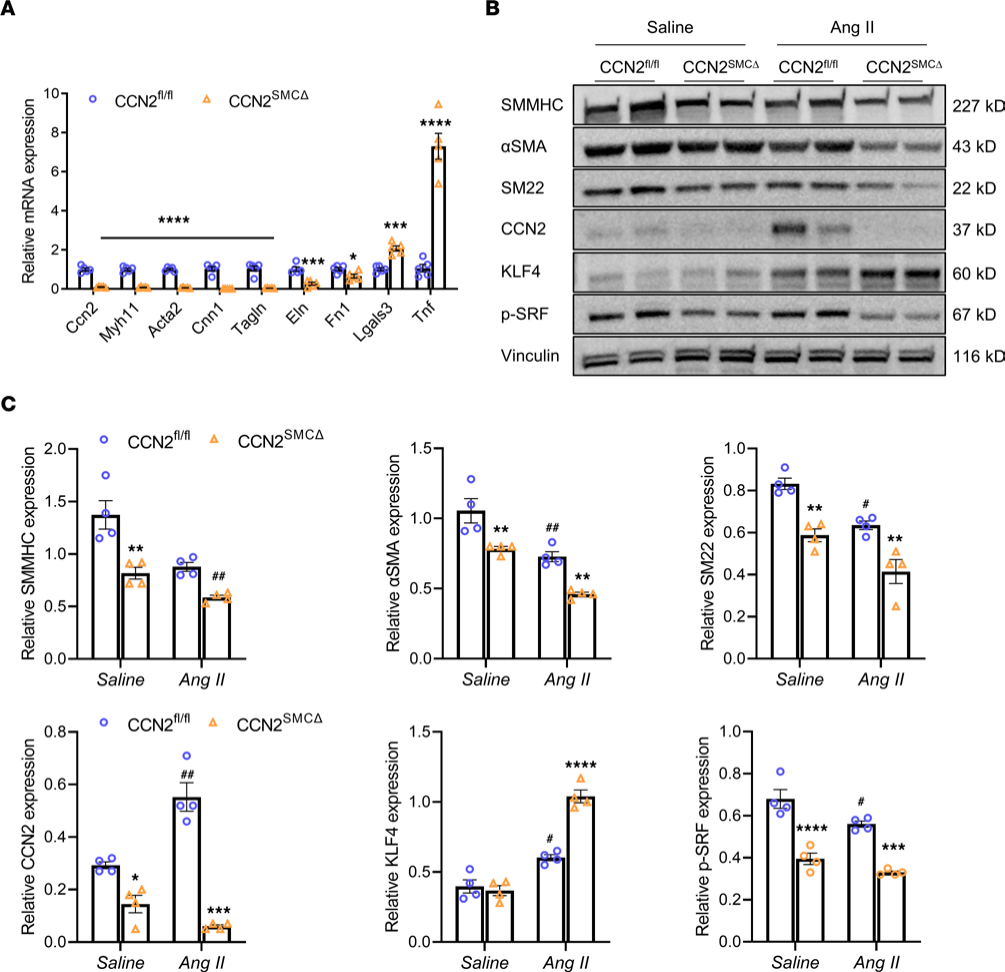
SMC-specific deficiency of CCN2 represses SMC contractile markers in vivo. (**A**) qPCR analysis of various mRNA expression in abdominal aortic lysates from CCN2^fl/fl^ and CCN2^SMCΔ^ mice with 7-day Ang II infusion (*n* = 5). *P < 0.05, ***P < 0.001, ****P < 0.0001, 2-tailed Student’s *t* test. (**B**) Western blot analysis of various protein expression in abdominal aortic lysates from mice in each group (*n* = 4). (**C**) Quantification of protein expression in **B**. Target protein levels were normalized to Vinculin. Data were quantified and represented as mean ± SEM. *P < 0.05, **P < 0.01, ***P < 0.001, ****P < 0.0001 (effect of CCN2 deficiency within same treatment group); ^#^*P* < 0.05, ^##^*P* < 0.01 (effect of Ang II within genotype group); 2-way ANOVA.

To confirm the involvement of CCN2 deficiency–associated loss of SMC contractile markers, quantitative PCR (qPCR) analysis was performed to compare the mRNA levels of SMC markers in abdominal aortas of CCN2^^fl/fl^^ and CCN2^^SMCΔ^^ mice with 7-day Ang II infusion. Deficiency of CCN2 in SMC resulted in a greater than 90% reduction of total CCN2 mRNA in mouse aortas. Correspondingly, Myh11 (SMMHC), Acta2 (αSMA), CNN1 (Calponin1), and Tagln (SM22), which are markers of SMC, as well as Eln (Elastin) and Fn1 (fibronectin 1), were significantly downregulated in CCN2^^SMCΔ^^ mice. Not surprisingly, however, Lgals3 (a marker of cell activation) and Tnf (a marker of tissue inflammation) were upregulated in CCN2^^SMCΔ^^ mice (Figure 5A).

In a separate cohort of animals, abdominal aortas from CCN2^^fl/fl^^ and CCN2^^SMCΔ^^ mice infused with Ang II or saline were harvested for protein expression analysis. CCN2 protein is expressed at low levels at baseline in saline-treated WT mice, and its expression increased substantially under the effects of Ang II. Nevertheless, knockdown of CCN2 in SMC significantly reduced the protein content of CCN2 in mouse aortas in both groups but with greater changes in magnitude in the Ang II group (Figure 5, B and C). Along with the reduction in CCN2, SMC contractile markers SMMHC, αSMA, and SM22 were moderately reduced in saline-treated CCN2-KO mice; Ang II infusion notably suppressed SMC markers in WT mice; and the protein levels of SMC markers were further reduced in CCN2-KO mice. Interestingly, CCN2 deficiency in SMC was also associated with a reduction in phosphorylated serum response factor (p-SRF), a positive regulator of SMC gene transcription (34–36), in mouse aortas of both treatment groups. In contrast, KLF4, a known transcriptional repressor of SMC marker genes (37, 38), was markedly induced by Ang II, and its levels were further upregulated in conjunction with CCN2 deficiency (Figure 5, B and C).

**Figure 6:**
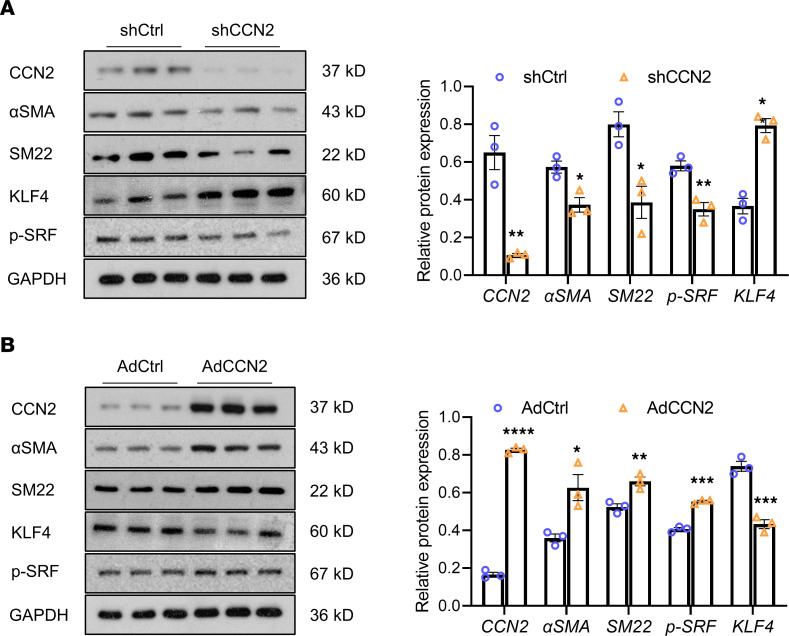
CCN2 regulates SMC contractile markers in vitro. Western blot analysis of various protein expression in cultured human primary aortic SMCs (HASMCs) with CCN2 deficiency (Ad-CCN2-shRNA versus Ad-scrmb-shRNA) (**A**) or overexpression (Ad-GFP-CCN2 versus Ad-GFP) (**B**). Target protein levels were normalized to GAPDH. Data were quantified and represented as mean ± SEM. *n* = 3. *P < 0.05, **P < 0.01, ***P < 0.001, ****P < 0.0001, 2-tailed Student’s *t* test.

To further decipher the biological effects of CCN2 in SMC specifically and to test the validity of our findings in vivo, HASMCs were cultured and infected with adenoviruses to either knock down or overexpress CCN2. In line with the in vivo findings, knockdown of CCN2 significantly reduced the protein contents of p-SRF and SMC markers — including αSMA, calponin, and SM22 — but increased the KLF4 transcription factor (Figure 6A). In contrast, CCN2 overexpression substantially augmented the levels of p-SRF and SMC markers but reduced KLF4 (Figure 6B). Our data suggest that CCN2 is critical in maintaining an SMC contractile phenotype both in vivo and in vitro, and they show that deficiency of CCN2 leads to the loss of contractile markers in SMC and predisposes those mice to aneurysm development following vascular injury.

### CCN2 interacts with and potentiates TGF-β1 receptor signaling in HASMCs.

**Figure 7:**
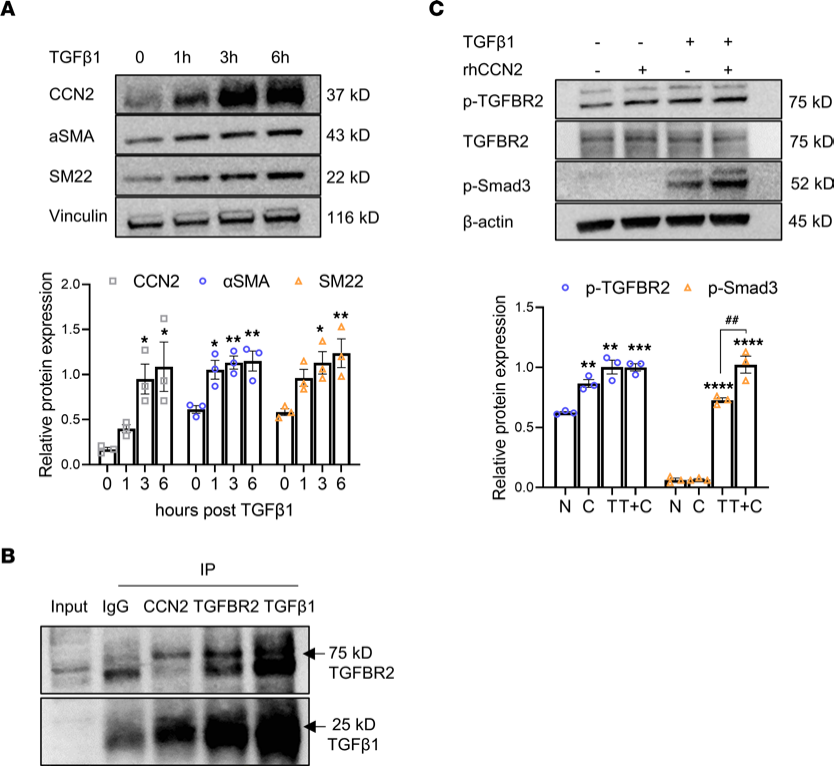
CCN2 interacts with and potentiates TGF-β1 receptor signaling. (**A**) TGF-β1 (1 ng/mL) induces protein expression of CCN2, αSMA, and SM22 in HASMCs by Western blot (*n* = 3). Target protein levels were normalized to Vinculin. *P < 0.05, **P < 0.01, compared with time point 0, using 1-way ANOVA. (**B**) Validation of physical interaction between CCN2 and TGF-β1/TGF-β receptor II via Co-IP followed by Western blot. Column 3 indicate positive bands for TGFBR2 (75 kD) and TGF-β1 (25 kD) in sediment precipitated with anti-CCN2 antibody; column 1, 20% input; column 2, IgG (negative control); column 4, TGFBR2 (positive control); column 5, TGF-β1 (positive control). (**C**) Supplementation of recombinant human CCN2 (rhCCN2) increases TGF-β receptor II phosphorylation and potentiates TGF-β1–induced Smad-3 phosphorylation in HASMCs by Western blot (*n* = 3). Target protein levels were normalized to β-actin. **P < 0.01, ***P < 0.001; ****P < 0.0001 (compared with nontreatment [N] group); ^##^*P* < 0.01 (compared with TGF-β1-only [T] group); 1-way ANOVA.

**Figure 8:**
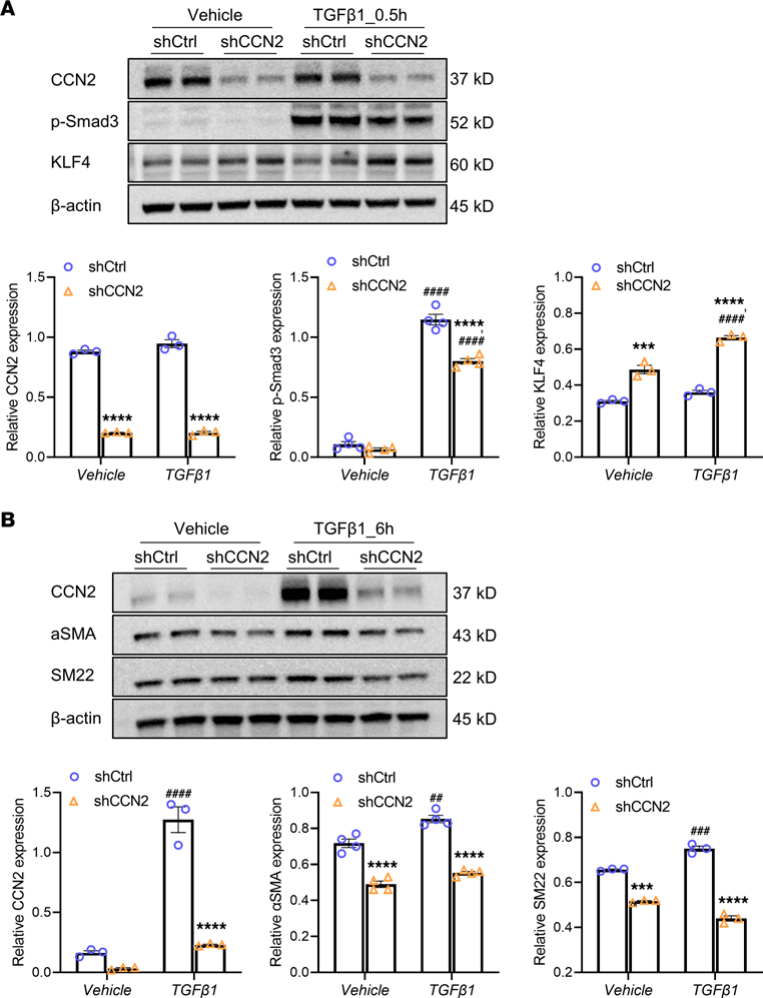
Deficiency of CCN2 attenuates TGF-β1–mediated signaling and SMC markers in vitro. (**A**) CCN2 deficiency attenuates TGF-β1–induced (0.5 hours) Smad3 phosphorylation and augments KLF4 expression in HASMCs. (**B**) CCN2 deficiency attenuates the expression of SMC contractile markers with and without TGF-β1 stimulation (6 hours) in HASMCs. Target protein levels were normalized to β**-**actin. Data were quantified and represented as mean ± SEM. *n* = 3. ***P < 0.001, ****P < 0.0001 (effect of CCN2 deficiency within same treatment group); ^##^*P* <0.01, ^###^*P* < 0.001, ^####^*P* < 0.0001 (effect of TGF-β1 within same genotype group); 2-way ANOVA.

As an ECM protein, CCN2 conveys its signaling potential through interaction with an array of receptors, ECM components, and growth factors. Specifically, studies have demonstrated that CCN2 is an essential cofactor for TGF-β and could potentiate TGF-β activity (5, 39). Considering the well-reported role of TGF-β signaling in SMC phenotype regulation in conjunction with RNA-Seq results that point to the involvement of the Smads and the receptor tyrosine kinase pathway associated with SMC CCN2 deficiency, we decided to take a deep look at the interplay between CCN2 and TGF-β. In cultured HASMCs, an intermediate dose of TGF-β1 at 1 ng/mL markedly stimulated CCN2 protein expression as early as 1 hour after treatment. Correspondingly, significant increases in αSMA and SM22 protein levels were also observed (Figure 7A). We then performed a co-IP assay in CCN2-overexpressing HASMCs to determine whether CCN2 could physically interact with TGF-β1 and/or the TGF-β receptor. After targeted pull-down with anti-CCN2 antibody, along with various positive and negative assay controls, the blots clearly showed the presence of both TGF-β1 and TGF-β receptor 2 in a CCN2 protein complex (Figure 7B). To test whether CCN2 could mediate TGF-β1 activity, HASMCs were incubated with recombinant human CCN2 (rhCCN2) with or without a low dose of TGF-β1 (0.1 ng/mL) for 30 minutes. The data demonstrate that rhCCN2 alone is capable of stimulating TGF-β2 receptor phosphorylation to some degree, yet it failed to evoke downstream Smad3 phosphorylation. However, in the presence of TGF-β1, a supplement of rhCCN2 significantly increased the phosphorylation of Smad3 (Figure 7C). Conversely, deficiency of CCN2 led to significant increases in KLF4 in both vehicle and TGF-β1–treated HASMCs. Moreover, TGF-β1–mediated Smad3 phosphorylation was markedly reduced in CCN2-deficient cells (Figure 8A). In line with the previous observation (Figure 7A), extended TGF-β1 stimulation (6 hours) significantly increased CCN2, αSMA, and SM22 protein levels; however, the stimulative effects of TGF-β1 on SMC marker expression were significantly blunted with CCN2 deficiency in HASMCs (Figure 8B). Together, these data suggest that CCN2 is both a target and mediator of TGF-β signaling and at least partially accounts for TGF-β1–mediated SMC contractile protein expression.

### TGF-β receptors are required for CCN2-mediated SMC contractile marker expression.

**Figure 9:**
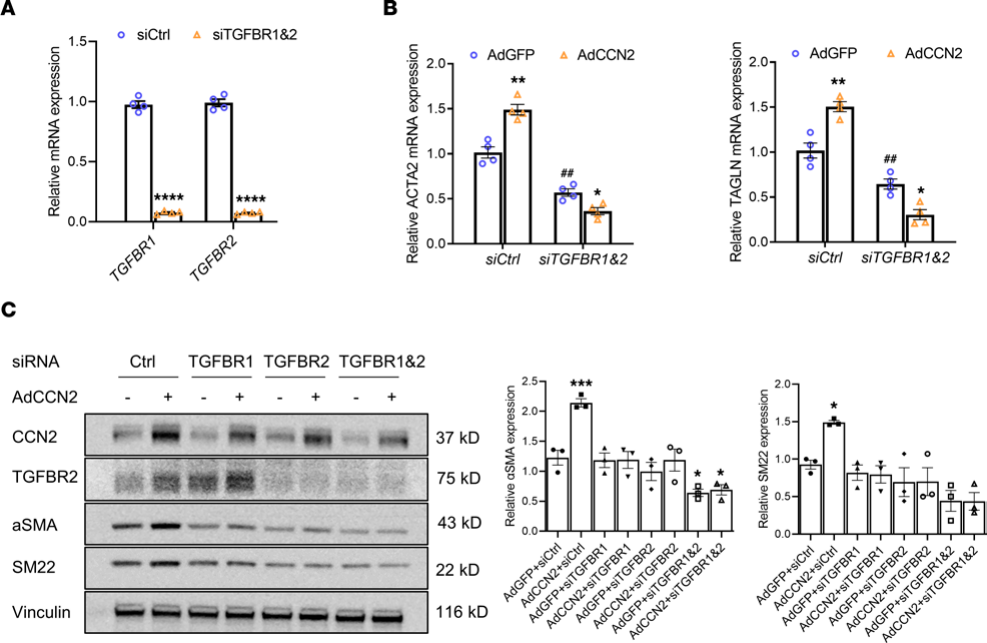
TGF-β receptors are required for CCN2-mediated SMC contractile markers expression. (**A**) Validation of siRNA-mediated knockdown of TGF-β receptors (TGFBR1, TGFBR2) by qPCR. *n* = 4. ****P < 0.001, 2-tailed Student’s *t* test. (**B**) Knockdown of TGF-β receptors blunted the stimulating effect of CCN2 overexpression on ACTA2 and TAGLN mRNA by qPCR. *n* = 4. *P < 0.05, **P < 0.01 (effect of CCN2 overexpression within same siRNA group); ^##^*P* < 0.01 (effect of siRNA treatment within same Ad group); 2-way ANOVA. (**C**) Knockdown of TGF-β receptors blunted the stimulating effect of CCN2 overexpression on αSMA and SM22 protein by Western blot. *n* = 3. Target protein levels were normalized to Vinculin. *P < 0.05, ***P < 0.001, 1-way ANOVA.

To explore the involvement of TGF-β receptors in CCN2-mediated regulation of SMC marker expression, we knocked down TGFBR1 and TGFBR2, either individually or together in HASMCs, followed by adenovirus-mediated overexpression of CCN2. Knockdown of TGF-β receptors using siRNA was verified by qPCR, which indicated over 90% efficiency (Figure 9A). While we consistently observed the induction of SMC markers, both mRNAs (Figure 9B) and proteins (Figure 9C), in CCN2 overexpressing cells, CCN2-mediated increases in αSMA (ACTA2) and SM22 (TAGLN) were eliminated in cells deficient of TGFBR1, TGFBR2, or both (Figure 9, B and C). Taken together, these data indicate that CCN2-mediated SMC contractile marker expression is likely dependent on TGF-β receptor involvement.

## Discussion

The results of this study are the first to our knowledge to demonstrate that the loss of SMC-specific CCN2 exacerbates aneurysm development in mice, both in the presence and absence of hypercholesterolemia. Aneurysms formed in CCN2-deficient mice were extensive and tortuous, with a focal infrarenal preference (Figure 2A). Tissue characterization of the aneurysm sites reveals severe media deterioration, elastin breakdown, immune cell infiltration, and lipid accumulation — features closely reminiscent of advanced human aortic aneurysm (Figure 2F and Supplemental Figure 1B). Intriguingly, resistance of C57BL/6 to Ang II–induced AAA is completely lost upon silencing of VSMC–specific CCN2. In a majority of murine AAA studies reported, hypercholesterolemia is required for successfully modeling AAA using Ang II (40), either by genetic ablation of apolipoprotein E (*Apoe^–/–^*) (41) or LDL receptor (*Ldlr^–/–^*) (42). Our acute studies revealed that Ang II alone (as short as 3 days) is sufficient to induce AAA at the infrarenal site in CCN2^^SMCΔ^^ mice but not in the controls (Figure 3). These findings clearly underscore SMC-derived CCN2 as a key ECM component of the vascular wall essential for the maintenance of vascular integrity and the prevention of structural damages, especially in response to injury.

Formation of AAA in the infrarenal aortic region of CCN2^^SMCΔ^^ mice in response to Ang II infusion is an intriguing finding. To the best of our knowledge, since the introduction of the Ang II model for AAA studies in mice, studies over the last 2 decades have reported AAA pathology at the suprarenal aortic region, a location different than the infrarenal aorta where human AAA occurs (40, 43). While our understanding of the pathology of AAA formation has grown substantially due to experiments derived from murine models of AAA that recapitulate several major features of human AAA, there is still a lack of progress in developing effective medical therapy for AAA, indicating that our current understanding of the pathophysiology of human AAA remains inadequate. Thus, new models that closely mimic human AAA are strongly desired. Aside from the obvious limitations of the use of murine species, which the research community relies upon to glean insights into pathophysiological mechanisms that may or may not translate into human AAA disease, the disparity in the anatomic locations of murine and human AAA is another major limitation. While it is conceivable that humans being biped while mice are quadruped could render regional hemodynamic and structural differences between human and mice that might account for the AAA location difference, the exact underlying mechanisms are unknown. Nevertheless, our findings of profound infrarenal AAA pathology in both Ang II and elastase models not only unequivocally demonstrate an unanticipated antianeurysmal role of SMC-CCN2, but also support that CCN2 mutant mouse lines may serve as a novel model for studying infrarenal AAA disease.

An important point to note is that our studies were carried out in the context of inducible deficiency of CCN2 in adult mice. Whether germline loss of SMC-CCN2 has any impact on vessel integrity, on vascular homeostasis, or in murine aortic aneurysm is a subject of future investigation. The proaneurysmal phenotype of CCN2^^SMCΔ^^ mice is corroborated by a recent study using whole-body CCN2-deficient mice (inducible) in which augmented aneurysm development in both thoracic and abdominal aorta with Ang II infusion was observed (44). Despite all the structural and molecular changes associated with CCN2 deficiency, change in VSMC function was not a particular focus in that study. Interestingly, data from previous a study implied that hemizygous KO of CCN2 is protective from elastase-induced AAA (45), findings that appear in disagreement with our results. However, those experiments were performed in constitutive whole-body CCN2^^+/–^^, a condition that is very different from our current approach, where SMC-CCN2 ablation is induced in the adult mice. It is likely that, in comparison with inducible loss of SMC-CCN2 in adult mice, the constitutive and systemic deficiency of CCN2 could trigger distinct mechanisms leading to developmental changes that might render an overall antianeurysm effect in the elastase model. Also, in light of the importance of all vascular wall intrinsic cell types (EC, SMC, and fibroblasts) and hemopoietic lineage cells in AAA pathology, it is possible that haploinsufficiency of CCN2 in different cellular compartments might have differential responses in the elastase-induced AAA model, an issue that can only be addressed with additional cell lineage–restricted KO animals.

Studies herein focused on SMC-CCN2, and this is in part attributed to the central importance of this cell type in the pathobiology of AAA. In support of this, our data show that SMC-CCN2 limits MMP activity, inflammation, and cellular apoptosis and preserves the SMC contractility, culminating in an overall protection against AAA (Figure 3, E–I; Figure 5; and Supplemental Figure 2B). However, we are not excluding the possibility that CCN2 produced by other cell types such as endothelial cells, adventitial fibroblasts, or immune cells may affect AAA — a possibility that necessitates follow-up studies in animals with restricted deficiency in the cell types mentioned above.

Contradictory to its profibrotic role in fibroblasts, CCN2 deficiency in SMC results in reduced elastin mRNA but increased breakage in aneurysm tissue that could compromise the overall strength of the vascular wall (Figure 3D, Figure 5A, and Supplemental Figure 2A). Interestingly, when elastin was eliminated via elastase and BAPN, the AAA phenotype in CCN2^^SMCΔ^^ mice was still significantly more severe than that in control mice, suggesting that CCN2 deficiency in SMC mediates deleterious effects beyond elastin dysregulation (Supplemental Figure 5). One important mechanism that likely contributes to the observed AAA phenotype in CCN2^^SMCΔ^^ mice is the reduction in SMC contractile marker expression. In the chronic Ang II setting, signals of SMMHC and αSMA are significantly diminished in the media layer of aortas at the aneurysm site in CCN2^^SMCΔ^^ mice compared with those in both the aneurysm and nonaneurysm sites in CCN2-floxed control mice, henceforth CCN2^^fl/fl^^ mice (Figure 2G and Supplemental Figure 1C). Similar reductions in SMC markers were also evident in the acute setting (Figure 5, A and B). Kyoto Encyclopedia of Genes and Genomes (KEGG) and Gene Ontology (GO) analyses with Metascape (https://metascape.org/gp/index.html#/main/step1) of the RNA-Seq data reveal that vascular smooth muscle contraction is one of the most affected pathways in SMC-CCN2–deficient aortas (Figure 4C). Moreover, cells within the aberrantly remodeled vessel wall show minimal SMC markers, and they were CD68^^–^^ (marker of macrophage lineages) and positive for vimentin and collagen I (markers for mesenchymal-like cells). This represents a unique cell population distinct from conventional SMCs (Figure 3, G–I). In addition, increases in cell proliferation and apoptosis were observed in that population, suggesting a synthetic phenotype with greater cell turnover (Figure 3, E and F). This unique cell population likely contributes to the increased MMP activity and elastin disruption in the aortas of the CCN2^^SMCΔ^^ mice (Supplemental Figure 2). These findings, while not against the prevailing view that VSMCs undergo a phenotypic switch from contractile to synthetic states in vascular diseases, coincide with a trending view that an array of SMC with diverse fates are present in vascular disease, as revealed by accumulating lineage tracing and single-cell RNA-Seq studies (46–50). Additional lineage-tracing studies are required in order for researchers to completely understand SMC fate due to CCN2 loss in murine AAA models.

As a matricellular protein, CCN2 binds to multiple receptors and growth factors and mediates crosstalk between the ECM and a variety of cells. In tissue fibrosis, it has been suggested that CCN2 partially mediates the profibrotic effects of both TGF-β and Ang II (3, 27, 51). Studies have shown that, in fibroblasts, TGF-β1–mediated induction of CCN2 mRNA occurs within 30 minutes of TGF-β treatment (22), and this effect is dependent on Smad3 signaling (52). This transcriptional regulation of CCN2 by TGF-β is further supported by studies showing the presence of a unique sequence located in the CCN2 promoter upon which Smad3 and Smad4 are able to bind (52). In addition, studies using biomolecular techniques demonstrated that CCN2 may function as a chaperone to facilitate TGF-β1/TGF-β receptor interaction and potentiate TGF-β1–mediated Smad activation (5). Here, our in vitro results echo previous findings and advance the knowledge regarding the interplay between CCN2 and TGF-β signaling in SMCs by providing evidence that (a) CCN2 is a target of TGF-β signaling and that TGF-β1–mediated induction of CCN2 protein is fast and robust (Figure 7A); (b) CCN2 can physically interact with the TGF-β1–TGF-β receptor complex and potentiate TGF-β1–mediated Smad3 phosphorylation (Figure 7, B and C); and (c) CCN2 and TGF-β signaling are mutually dependent on maintaining the contractile function of SMC, as deficiency of either CCN2 or TGF-β receptors suppress SMC markers (Figures 8 and 9).

Concordant with the intricate functional relationship between CCN2 and TGF-β, mice with SMC-specific ablation of TGF-β signaling on a proatherosclerotic background (either *Apoe^–/–^* or *Ldlr^–/–^*) developed aortic aneurysms in response to a hypercholesterolemic diet (21). Moreover, the extensive and tortuous presentation of the aneurysms is very similar to those observed in our chronic Ang II model in CCN2^^SMCΔ^^ mice. Mechanistically, the data suggest that a combination of medial SMC loss with increases in transdifferentiation of contractile SMCs to MSC-like cells contribute to aneurysm development in those mice. In addition, an elevated expression of KLF4, a direct target suppressed by TGF-β, was observed in the mouse aortas with either CCN2 or TGF-βR2 deficiency in SMCs (21). Therefore, it is tempting to speculate that augmented aneurysm development in SMC-specific TGF-βR2 deficient mice might partially attribute to the disruption of signaling normally mediated by SMC-derived CCN2. To gain in-depth insight into the functional importance of CCN2 for TGF-β pathway or vice versa in AAA, it would be interesting to know whether CCN2 expression/function is altered in SMC–TGF-βR2–KO mice; additional in vivo studies to determine whether CCN2 sufficiency or deficiency affects the AAA phenotype in animals deficient in SMC–TGF-βR2 are required. Together, our studies point to the potential importance of the cooperation of CCN2 and the TGF-β pathway in vascular homeostasis, an exciting topic that merits further interrogation.

In summary, our findings demonstrate that deficiency of SMC-specific CCN2 alters SMC phenotype and function to result in compromised vascular integrity and aneurysm development. Owing to the uniqueness of intrarenal AAA pathology in CCN2^^SMCΔ^^ mice, our studies suggest that CCN2^^SMCΔ^^ mice might serve a tool to gain knowledge in disease mechanisms for human AAA. Finally, it is exciting to envision the perceived requirement of CCN2 for TGF-β action and vice versa for normal vascular homeostasis in general and in AAA disease, in particular.

## Methods

### Animals

CCN2-floxed mice (originally acquired from Andrew Leask, The University of Western Ontario, London, Ontario, Canada) were crossed with Myh11–Cre-ER^^T2^^ transgenic mice (The Jackson Laboratory, strain no. 019079) to generate male CCN2-floxed mice harboring SMC-specific Cre (CCN2^^fl/fl^^–Myh11–Cre-ER^^T2^^). SMC-specific deletion of CCN2 was achieved by i.p. injection of 5 consecutive doses of tamoxifen (0.1 mL at 20 mg/mL dissolved in sunflower seed oil; T5648, Sigma-Aldrich) to 8-week-old maleCCN2^^fl/fl^^–Myh11–Cre-ER^^T2^^ mice. Age-matched male CCN2-floxed mice without Cre expression subjected to the same tamoxifen regimen were used as controls. After a 2-week washout period, CCN2^^SMCΔ^^ mice and CCN2^^fl/fl^^ mice were used for the modeling of AAA.

#### Ang II infusion model.

For the long-term Ang II model, tamoxifen-injected CCN2^^fl/fl^^ (*n* = 13) and CCN2^^SMCΔ^^ mice (*n* = 11) were subjected to a single injection of adeno-associated virus overexpressing a mouse form of PCSK9 (1 × 10^^11^^ VG, AAV8-D337y-mPCSK9, Vector Biolabs) via tail vein and were fed a high-fat diet (D12108C, Research Diets) for the duration of the studies. Two weeks after PCSK9 injection, Ang II was systemically infused by ALZET miniosmotic pumps (Model 2006, ALZET) as previously described (53) at the dose of 500 ng/kg/min (A9525, Sigma-Aldrich) for 42 days. For short-term Ang II infusion, tamoxifen-injected CCN2^^fl/fl^^ (*n* = 18) and CCN2^^SMCΔ^^ mice (*n* = 21) only received Ang II infusion via miniosmotic pumps (Model 1007D, ALZET) at the dose of 500 ng/kg/min for 7 days. Additional CCN2^^fl/fl^^ (*n* = 10) and CCN2^^SMCΔ^^ mice (*n* = 10) receiving saline-filled osmotic pumps were used as saline controls.

#### BAPN-elastase model.

The 0.2% BAPN (A3134, Sigma-Aldrich) containing drinking water (in light-shield red bottles) was provided to mice 2 days before surgery until the end of the studies. BAPN containing drinking water was freshly prepared and changed every other day. Tamoxifen-injected CCN2^^fl/fl^^ (*n* = 10) and CCN2^^SMCΔ^^ mice (*n* = 10) were subjected to the surgery for elastase exposure as described elsewhere (26). Briefly, surgeries were performed on mice under anesthesia to expose the lower infrarenal abdominal aorta. In total, 5 μL of porcine pancreas elastase (Sigma-Aldrich) was carefully applied to the exposed aortic adventitia at the bottom of the boat-shaped area and incubated for 5 minutes. The exposed area was gently washed afterward with normal saline, and surgical sites were carefully sorted and restored before closing. As for the control groups, additional CCN2^^fl/fl^^ (*n* = 5) and CCN2^^SMCΔ^^ mice (*n* = 5) were subjected to the same surgical procedure, except for the substitution of the active elastase with the heat-inactivated elastase (100°C for 30 minutes).

### In vitro studies

HASMCs were purchased from American Type Culture Collection (ATCC, PCS-100-012) and cultured in vascular cell basal medium (ATCC, PCS-100-030) supplemented with growth kit (ATCC, PCS-100-042). Cells at passage 5–7 were used for various studies. Adenoviruses used for CCN2 overexpression (Ad-GFP-hCTGF, ADV-206232) and knockdown (Ad-GFP-U6-h-CTGF-shRNA, shADV-206232) studies, henceforth AdCCN2 and shCCN2, and control viruses (Ad-GFP, Ad-GFP-U6-scrmb-shRNA), henceforth AdCtrl and shCtrl, were purchased from Vector Biolabs. siRNAs used for human TGF-β receptor knockdown (TGFBR1-7046, TGFFBR2-7048, and Nontargeting Pool) were purchased from Horizon. rhCCN2 (9190-cc) and TGF-β1 (rhTGF-β1, 240-B/CF) were purchased form R&D Systems and reconstituted in sterile PBS and 4 mM HCl-0.1% BSA, respectively, as recommended by the manufacturer.

For CCN2 knockdown– or overexpression-related studies, HASMCs at 80% confluence were infected with shCtrl, shCCN2, AdCtrl, or AdCCN2 at the multiplicity of infection (MOI) of 10. After overnight incubation, virus containing medium was replaced with fresh medium and sat for 48 hours, followed by 6-hour starvation (0.5% FBS) before harvesting or subsequent treatment. In one study, naive cells at confluence were fasted and treated with rhTGF-β1 (1 ng/mL) for 1, 3 and 6 hours to assess the expression of CCN2 and SMC markers. In another study, lower doses of rhTGF-β1 (0.1 ng/mL) and rhCCN2 (10 ng/mL) were used in combination to assess TGF-β receptor and Smad3 phosphorylation. In the context of CCN2 deficiency, short-term rhTGF-β1 exposure (1 ng/mL, 0.5 hours) and prolonged exposure (1 ng/mL, 6 hours) were performed to study the quick (phosphorylation) and slow (protein synthesis) responses respectively.

For Co-IP, HASMCs were infected with AdCCN2 and cultured for 66 hours, followed by 6-hour starvation (0.5% FBS). Cells were then incubated with rhTGF-β1 (1 ng/mL) and rhCCN2 (100 ng/mL) for 30 minutes. After that, culture medium was completely removed, and cells were rinsed with 5 mL HBSS (Corning) and fixed with 1% formaldehyde on ice for 10 minutes. After another rinse with HBSS, 1 mL of Co-IP buffer with Halt protease and phosphatase inhibitor cocktail (Thermo Fisher Scientific) was added to each 100 mm dish, incubated on ice for 10 minutes, and cells were scraped and collected to 1.5 mL microcentrifuge tubes. Cell lysates were centrifuged at 10,000*g* (15 minutes, 4°C), and 500 μL clear supernatant was used for each i.p. injection using preprepared antibody-bound protein G beads (Santa Cruz Biotechnology Inc., sc-2002). Normal rabbit IgG (Sigma-Aldrich, I5006) in substitution of anti-CCN2 antibody was included as a negative assay control, while additional precipitations using anti–TGF-β1 and anti-TGFBR2 antibodies were used as positive controls. Detailed antibody information is provided in Supplemental Table 2.

For the siRNA-mediated TGF-β receptor knockdown study, HASMCs were cultured in a 6-well culture plate until subconfluent (70%–80% confluency) and transfected with target siRNAs (siTGFBR1, siTGFBR2, siTGFBR1 + siTGFBR2) or control siRNA with lipofectamine RNAiMAX reagent (Invitrogen, 56532) at the dose of 30 nM. Six hours After siRNA transfection, medium was changed, and cells were infected overnight with either AdCtrl or AdCCN2 and cultured for an additional 48 hours before harvesting.

For cell apoptosis studies, control (shCtrl) or CCN2-deficient (shCCN2) HASMCs (shRNA-mediated gene knockdown) were treated with vehicle (DMSO) or 1 μM Staurosporine (AdipoGen, AG-CN2-0022-M001) for 6 hours and subjected to the Western blot analysis of Caspase 8 (cleaved) and Caspase 3 (cleaved). In vitro TUNEL assay was performed on control and CCN2-deficient HASMCs cultured on cover glasses and treated with vehicle (DMSO) or 1 μM Staurosporine for 3 hours using an In Situ Cell Death Detection Kit, TMR red (Roche) per manufacturer instructions.

### Aortic ultrasound imaging

Abdominal aortas were visualized in isoflurane-anesthetized mice at baseline and various time point after treatment and at study endpoints using the portable Vevo 3100 Micro-Imaging System with a D550 probe (FUJIFILM). Longitudinal and cross-sectional images of the abdominal aortas were captured and normal and maximal diameters of the abdominal aortas at aneurysm sites were measured. All recordings were made by 2 researchers in a blinded manner.

### Histochemistry and immunofluorescence staining

At study endpoints, mice were euthanized via isoflurane overdose and aorta segments were carefully isolated and harvested. For histochemistry, aorta segments were rinsed in ice-cold PBS, fixed in 10% buffered formalin for less than 24 hours, and transferred to 70% ethanol for paraffin embedding. Serial sections (5 μm sections) from each group were deparaffinized and used for morphological study by H&E staining and for visualization of elastin fibers assessed by Verhoeff-van Gieson staining using the Elastic Stain Kit (Abcam, ab150667). Images were acquired using NanoZoomer (Hamamatsu) at 20× resolution and processed by NDP.veiw 2. For immunofluorescence staining, aorta segments were rinsed in ice-cold PBS, directly embedded in OCT compound (Sakura, 4583), and slowly frozen down to solid. Cryosections (5 μm sections) from each group were air-dried, washed, and briefly fixed with 10% PBS-buffered formalin for 10 minutes. Aortic sections were then permeabilized with 0.1% Triton X-100 for 10 minutes and blocked with 10% BSA for 30 minutes at room temperature (RT). Primary antibodies (αSMA, SM-MHC, Ki67, and CD68; see Supplemental Table 2 for specific details) at 2 μg/mL were prepared in 1% BSA, added to each section, and incubated overnight at 4°C. Sections were then incubated with host-specific secondary antibodies with Alexa Fluor 488 or 594 (2 μg/mL) for 2 hours at RT protected from light. DAPI at 1 μg/mL was used to counterstain the nuclei. Fluorescence signals were visualized, and images were captured using a fluorescence microscope (Olympus IX71).

### In situ cell death (TUNEL)

Cell apoptosis was detected on freshly cut OCT-embedded abdominal aorta sections (5 μm) from CCN2^^fl/fl^^ and CCN2^^SMCΔ^^ mice (*n* = 5 per group) infused with saline or Ang II for 7 days using a In Situ Cell Death Detection Kit, Fluorescein (Roche), per manufacturer’s instructions. Briefly, DNA breaks were labeled at the free 3’-OH termini, with modified nucleotides (fluorescein-dUTP), during enzymatic reaction in cells, underwent apoptosis; green fluorescence signals were visualized; and images were captured by fluorescence microscopy. Negative controls were performed on parallel sections in the absence of enzyme solution.

### In situ MMP activity

In situ MMP activity was performed on freshly cut OCT-embedded abdominal aorta sections (5 μm) from CCN2^^fl/fl^^ and CCN2^^SMCΔ^^ mice (*n* = 5 per group) infused with saline or Ang II for 7 days using a Gelatinase/Collagenase Assay Kit (Invitrogen, E12055) per manufacturer instructions. Briefly, fluorescein-conjugated gelatin substrate was prepared and applied to sections and allowed to incubate at RT for 36 hours. Green fluorescence develops in the presence of enzymatic activities and was examined using a fluorescence microscope. Negative controls were performed on parallel sections in the presence of 5 mM EDTA.

### RNA extraction and qPCR

Total RAN was isolated using the RNase Mini Kit (Qiagen). RNA concentrations were determined using a Nanodrop One^^c^^ spectrophotometer (Thermo Fisher Scientific). In total, 100 ng of total RNA was used for reverse transcription to make cDNA using iScript Reverse Transcription kit (Bio-Rad, 1708841). Gene expression was assessed by SYBR green on QuantStudio 7 (Applied Biosystems). Data were normalized to GAPDH or β-actin using the ΔΔCt method. As a standard practice for qPCR experiments, no template and no RT controls were always included for quality control purposes. Detailed primer information is provided in Supplemental Table 1.

### Tissue lysis and Western blot

Snap-frozen aortic tissues were smashed in 1.5 mL microcentrifuge tubes using a pestle in liquid nitrogen. Tissues were homogenized in 100–200 μL ice-cold T-PER Tissue Protein Extraction Reagent (Thermo Fisher Scientific) supplemented with Halt protease and phosphatase inhibitor cocktail (Thermo Fisher Scientific) using a pellet pestle motor (Kimble-Kontes) and centrifuged at 10,000*g* (15 minutes, 4°C). For protein extraction of cultured cells, 200 μL per well (6-well plate) of ice-cold protein extraction reagent was directly added to cell culture dish after PBS wash and left on ice for 10 minutes. Cells with extraction buffer were scraped and collected into 1.5 mL Eppendorf tube and centrifuged at 10,000*g* (15 minutes, 4°C). In both cases, supernatants were collected, and protein concentrations were determined by BAC assay (Pierce). Equal amounts of resultant protein were run on 4%–20% SDS-PAGE gel under denaturing conditions, transferred to nitrocellulose membrane, and subjected to Western blot analyses using specific antibodies. Depending on the compatibility and availabilities of the antibodies, membranes were reprobed with antibodies against either β-actin, GAPDH, or Vinculin to confirm equal loading. Detailed antibody information is provided in Supplemental Table 2. Target proteins were detected by Immobilon Western HRP substrate (MilliporeSigma) and exposure (Kodak). Bands’ intensity was analyzed by ImageJ (NIH).

### RNA-Seq studies

Total RNAs extracted from abdominal aortas in mice infused with saline or Ang II for 7 days (4 groups, *n* = 3 per group) passed RNA sample quality control were subjected to commercial RNA-Seq (Novogene Corporation Inc.) using the NovaSeq PE150 platform at a depth of 6 G raw data per sample. Differentially expressed genes were identified by DEG-Seq based on *P* < 0.05 and |log__2__ fold change| > 1.5. Heatmap package (version 1.0.8, https://cran.r-project.org/web/packages/pheatmap/index.html) in R was utilized to perform the hierarchical cluster analysis. A volcano plot was generated to visualize up- and downregulated genes in CSA versus CFA aortic tissues. KEGG and GO enrichment were performed using Metascape (http://metascape.org/gp/index.html#/main/step1) to show the biological pathways and processes affected in the abdominal aorta associated with SMC-specific CCN2 deletion with Ang II infusion. RNA-Seq data have been deposited in the NCBI’s Gene Expression Omnibus (accession number GSE221399).

### Statistics

Data are presented as mean ± SEM. For data form 2 groups, an unpaired Student’s 2-tailed *t* test was performed. One-way ANOVA (followed by Tukey post hoc test) was performed to compare a single variable in multiple groups, and 2-way ANOVA (followed by Dunnett post hoc test) was used for 2-factor analysis. χ^^2^^ test was preformed to assess statistical significance for aneurysm incidence between 2 groups. Statistical analyses were performed using Prism 9.0 software. *P* < 0.05 was considered statistically significant.

### Study approval

All studies using mice were approved by an Institutional Animal Review Committee at Emory University and were conducted in accordance with the *Guide for the Care and Use of Laboratory Animals* (National Academies Press, 2011). Human aortic tissue samples were gifted by Guo-Ping Shi (Department of Medicine, Brigham and Women’s Hospital and Harvard Medical School, Boston, Massachusetts, USA). Frozen human AAA lesions and adjacent normal abdominal aortas were obtained from the Brigham and Women’s Hospital as previously reported (54). Discarded and decoded human aortas were reused according to the protocol 2010P001930 preapproved by the Human Investigation Review Committee at the Brigham and Women’s Hospital.

## Author contributions

The manuscript was written through contributions of all authors. All authors have given approval to the final version of the manuscript. YW, XL, and ZL designed the experiments. YW, XL, and WX performed the experiments. YW, XL, and QX analyzed the data. WX and XZ assisted with mouse breeding and tail vein injections. YW and ZL wrote the manuscript. ZL supervised the project. AL provided floxed-Ccn2 mouse strain. First authorship order position is based on intellectual contribution to design of the study and interpretation of data.

## Supplementary Material

Supplemental data

## Version 1 01/10/2023

Electronic publication

## Version 2 07/13/2023

Addition of author Andrew Leask
