# Supplementary material for: CCN2 deficiency in smooth muscle cells triggers cell reprogramming and aggravates aneurysm development
Source: JCI Insight. 2023 Jan 10;8(1):e162987. doi: 10.1172/jci.insight.162987 (PMC9870081; doi:10.1172/jci.insight.162987)
Supplement: Supplemental data [file jciinsight-8-162987-s010.pdf]

## Supplementary Materials for

# CCN2 deficiency in smooth muscle cells triggers cell reprogramming and aggravates aneurysm development

Yu Wang[1], Xuesong Liu[1,2], Qian Xu[1], Wei Xu[1], Xianming Zhou[1,3], Zhiyong Lin[1]

## Supplemental Figures

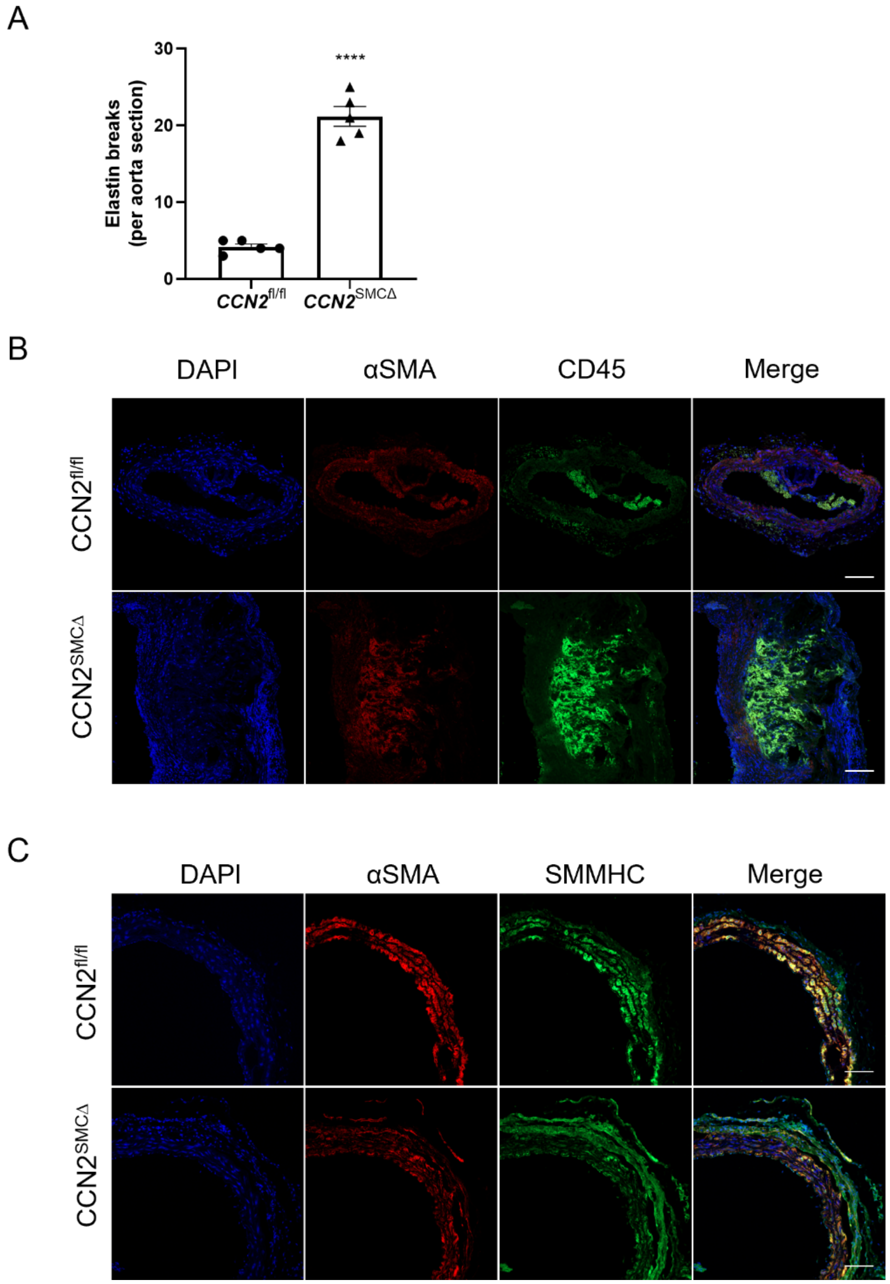


**Figure S1. (A)** Quantification of elastin breaks (counts per aorta section, 42-day Ang II infusion), n=5. Data were quantified and represented as mean ± SEM, ****, P<0.0001, 2-tailed Student's t test. **(B)** Immunofluorescence double staining of infrarenal aortic sections from CCN2$^{fl/fl}$ and CCN2$^{SMC\Delta}$ mice with specific antibodies against SM-α-actin (red) and CD45 (green). Nuclei were stained with DAPI (blue), n=3. **(C)** Immunofluorescence double staining of upper abdominal aortic sections (no dilations) from both groups with specific antibodies against SM-α-actin (red) and SMMHC (green). Nuclei were stained with DAPI (blue), n=3. Scale bars, 100 μm.

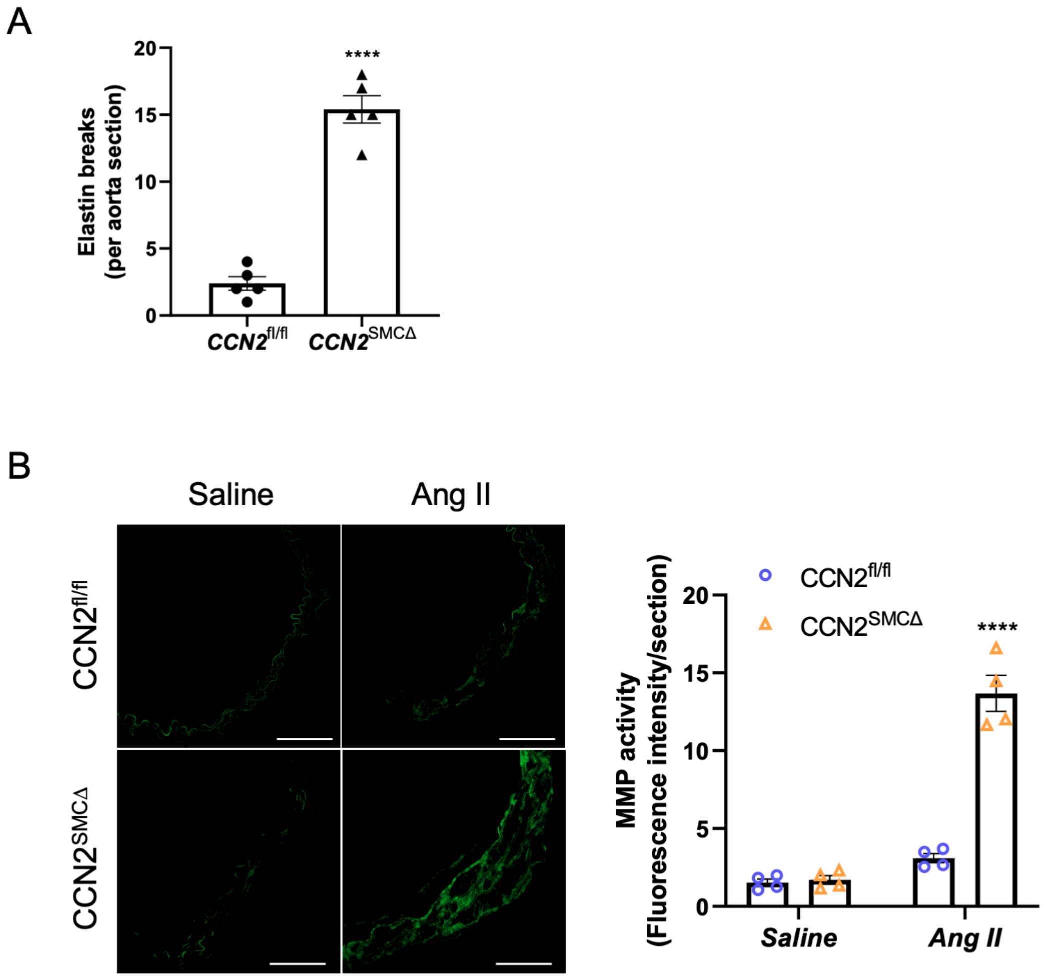


**Figure S2. (A)** Quantification of elastin breaks (counts per aorta section, 7-day Ang II infusion), n=5. **(B)** In situ zymography analysis of MMPs activity in infrarenal aortic sections from CCN2$^{fl/fl}$ and CCN2$^{SMC\Delta}$ mice infused with saline or Ang II (n=4). Total MMPs activity are indicated by positive green signals and quantified as fluorescence intensity per section. Scale bars, 100 μm. Data were represented as mean ± SEM, ****, P<0.0001, 2-way ANOVA.

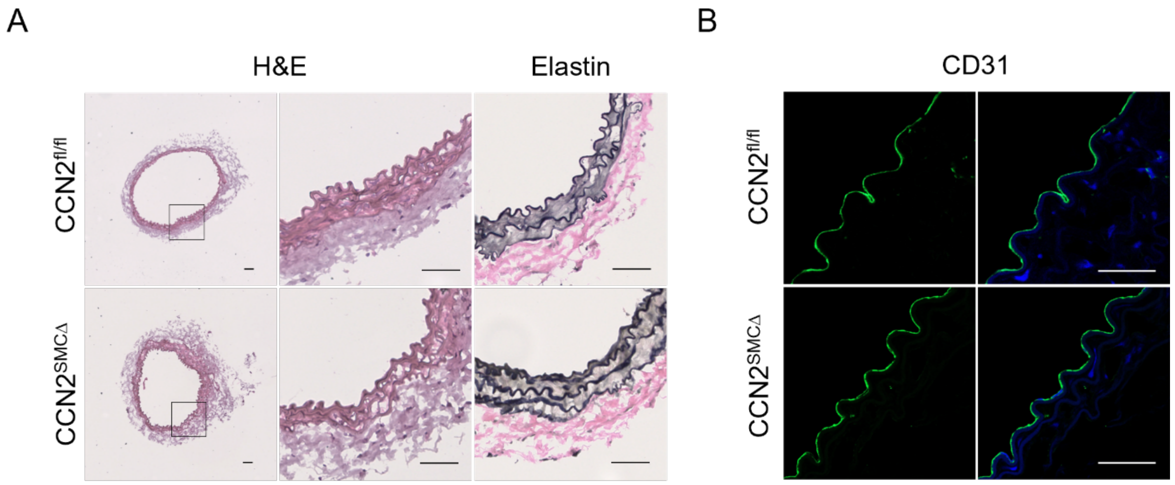


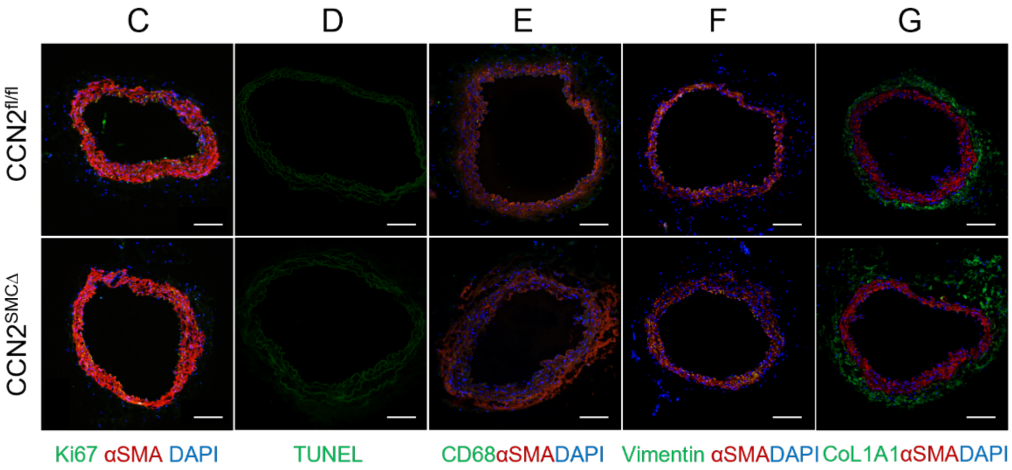


**Figure S3**. **(A)** Representative images of hematoxylin/eosin-stained (5x and 20x) and Elastin-stained (20x) abdominal aortic sections from saline- infused CCN2$^{fl/fl}$ and CCN2$^{SMC\Delta}$ mice (n=4). Scale bars, 100 μm. **(B)** Immunofluorescence staining of abdominal aortic sections from both groups with specific antibodies against CD31 (green). Nuclei were stained with DAPI (blue). Scale bars, 100 μm, n=4. Abdominal aortic sections (non-aneurysm) from saline- infused CCN2$^{fl/fl}$ and CCN2$^{SMC\Delta}$ mice stained with Ki67/αSMA **(C)**, TUNEL **(D)**, CD68/αSMA **(E)**, Vimentin/αSMA **(F)** and CoL1A1/αSMA **(G)**.

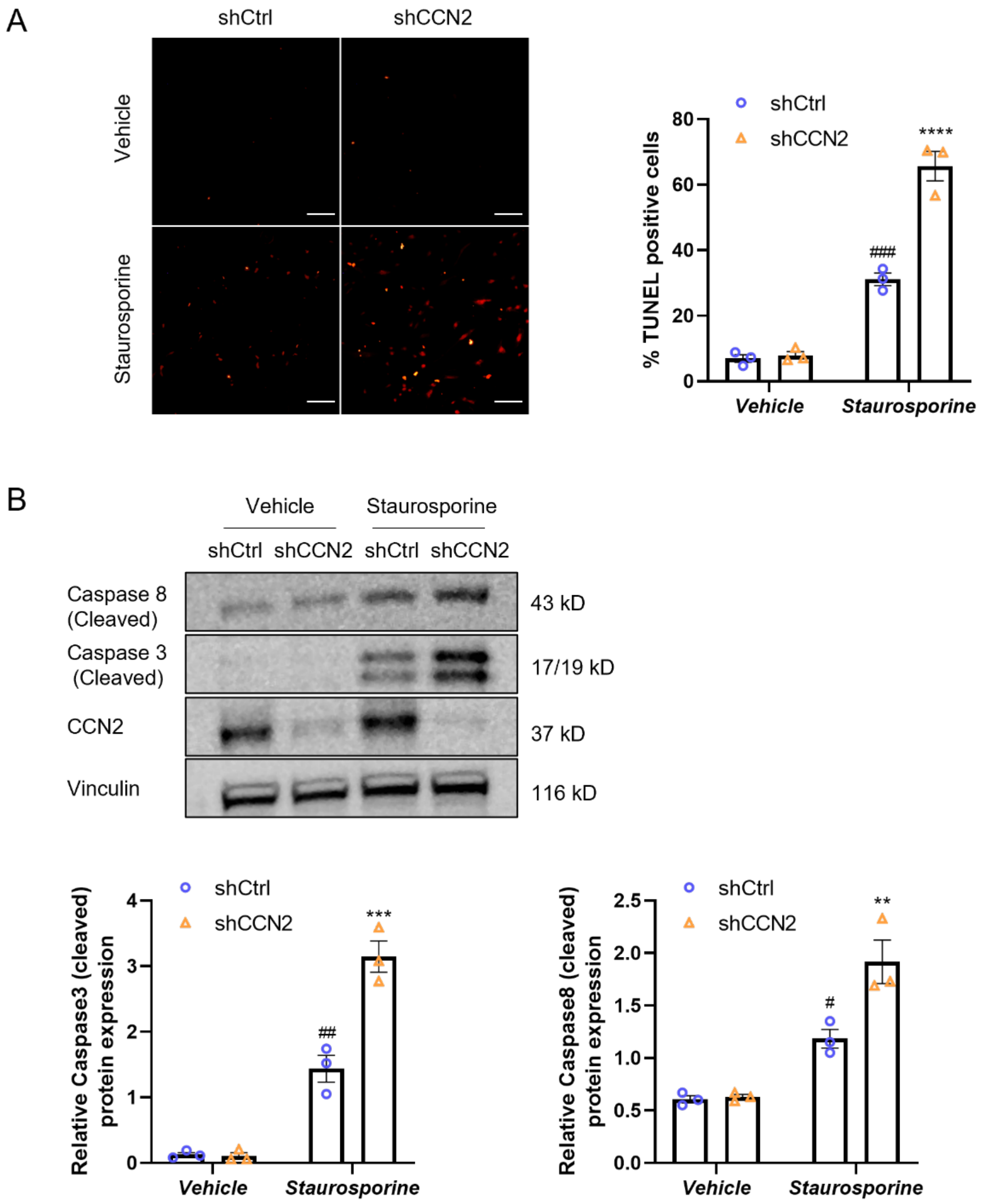


**Figure S4. SMC-specific deficiency of CCN2 exacerbates cell apoptosis in vitro. (A)** Representative images of TUNEL stained control and CCN2 deficient HASMCs treated with Vehicle or Staurosporine (n=3). Apoptotic cells were stained red. Scale bar, 100µm. **(B)** Western blot analysis of the active forms of caspase 8 and caspase 3 in control and CCN2 deficient HASMCs treated with Vehicle or Staurosporine (n=3). Target protein levels were normalized to vinculin. Data were quantified and represented as mean ± SEM. **, P<0.01, ***, P<0.001, ****, P<0.0001, effect of CCN2 deficiency within same treatment group; $^{\#}$, p<0.05, $^{\#\#}$, p<0.01, $^{\#\#\#}$, p< 0.001, effect of Staurosorine within same genotype group, 2-way ANOVA.

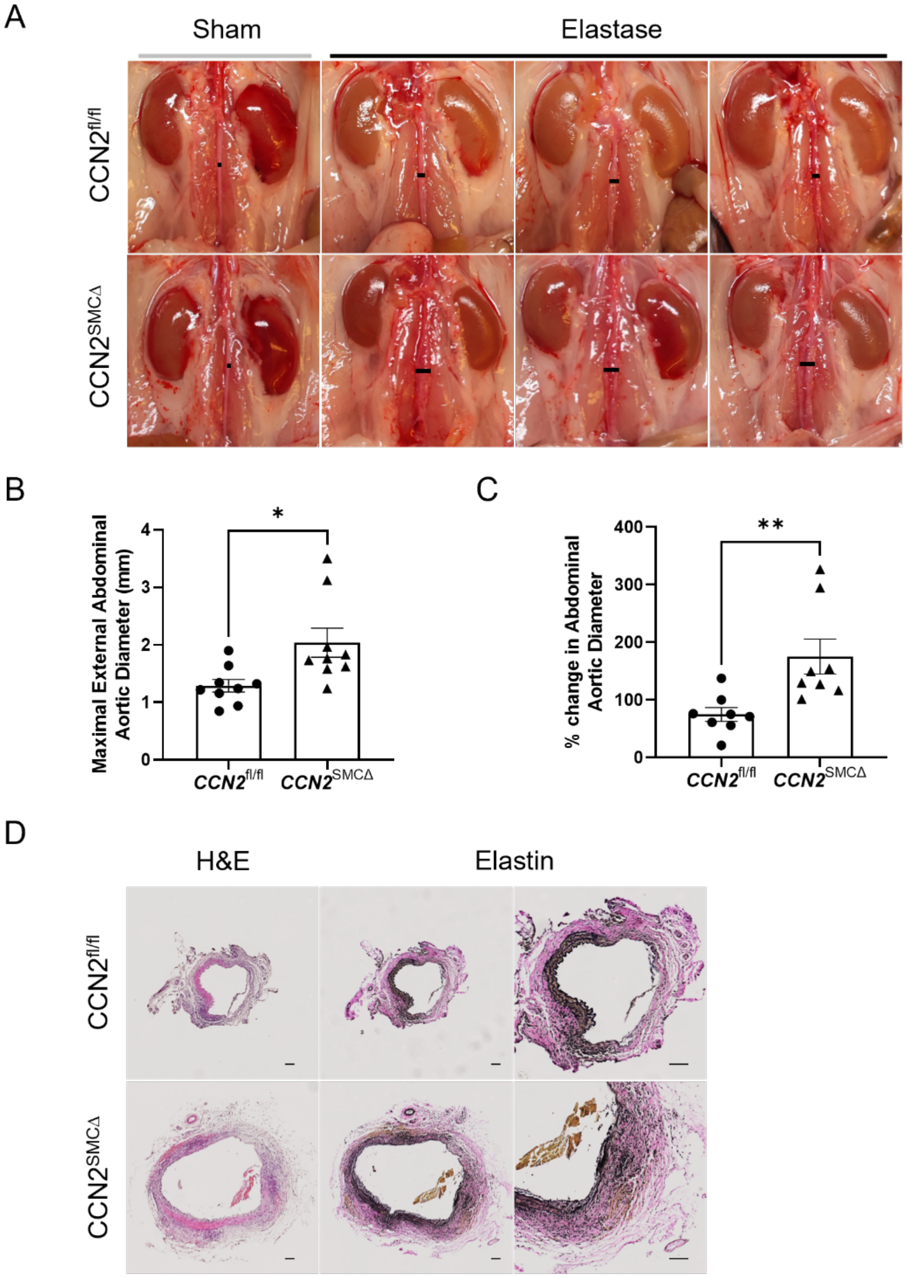


**Figure S5. Exacerbation of elastase-BAPN induced infrarenal aortic aneurysm in $CCN2^{SMC\Delta}$ mice. (A)** Representative gross images of abdominal aortas from sham-operated (n=3) and elastase-BAPN treated $CCN2^{fl/fl}$ and $CCN2^{SMC\Delta}$ mice (n=9). **(B)** Maximal external aortic diameters at study endpoint in mice from both groups. **(C)** % change in maximal external aortic diameters. Data were quantified and represented as mean ± SEM, *, P<0.05, **, P<0.01, 2-tailed Student's t test. **(D)** Representative images of H&E-stained (5x) and Elastin-stained (5x and 10x) abdominal aortic sections from both groups (n=5). Scale bars, 100 μm.

## Supplemental Tables

### Table 1. List of primers used

| **Gene Name (Mus)** | **Primer sequence (Forward 5' to 3')** | **Primer sequence (Reverse 5' to 3')** |
|---|---|---|
| Ccn2 | TGACCTGGAGGAAAACATTAAGA | AGCCCTGTATGTCTTCACACTG |
| Myh11 | GGGTAGCCAAGAAGCTGGTGT | TTTTCCACCAACTCCACGACC |
| Acta2 | CCCACCCAGAGTGGAGAA | ACATAGCTGGAGCAGCGTCT |
| Cnn1 | GAAGGTCAATGAGTCAACTCAGAA | CCATACTTGGTAATGGCTTTGA |
| Tagln (Sm22) | CCTTCCAGTCCACAAACGAC | GTAGGATGGACCCTTGTTGG |
| Eln | TGGAGCAGGACTTGGAGGT | CCTCCAGCACCATACTTAGCA |
| Fn1 | ATGTGGACCCCTCCTGATAGT | GCCCAGTGATTTCAGCAAAGG |
| Lgals3 | TCCTGGTTGAAGCTGACCAC | CGCTGGTGAGGGTTATGTCA |
| Tnf | AGTCCGGGCAGGTCTACTTT | GAGTTGGACCCTGAGCCATA |
| Gapdh | CATGGCCTTCCGTGTTCCTA | CCTGCTTCACCACCTTCTTGAT |
| Actb | CGCCACCAGTTCGCCATGGA | TACAGCCCGGGGAGCATCGT |
| **Gene Name (Homo)** | **Primer sequence (Forward 5' to 3')** | **Primer sequence (Reverse 5' to 3')** |
| TGFBR1 | GTGACAGATGGGCTCTGCTT | AAGGGCCAGTAGTTGGAAGT |
| TGFBR2 | GCACGTTCAGAAGTCGGATG | CTGCACCGTTGTTGTCAGTG |
| ACTA2 | GCGTGTAGCACCTGAAGAG | GAATGGCGACGTACATGGCA |
| TAGLN (SM22) | CACTGGCAGTTATAGGTGTTGAC | CCCACCCAGATTCATCAGCG |
| GAPDH | AGCCACATCGCTCAGACAC | GCCCAATACGACCAAATCC |

**Table 2. List of antibodies used**

| Target antigen | Vendor/Source | Catalog # | Working dilution |
|---|---|---|---|
| CCN2 | Cell signaling Technology | 10095 | 1:500 |
| SMMHC | Abcam | ab53219 | 1:500 |
| αSMA | Cell signaling Technology | 56856 | 1:1000 |
| SM22 | Cell signaling Technology | 40471 | 1:5000 |
| Calponin | Cell signaling Technology | 17819 | 1:500 |
| KLF4 | R&D Systems | AF3158 | 1:500 |
| p-SRF | Cell signaling Technology | 4261 | 1:500 |
| TGFβ1 | Abcam | ab92468 | 1:500 |
| TGFBR2 | Cell signaling Technology | 41896 | 1:500 |
| p-TGFBR2 | Abcam | ab183037 | 1:500 |
| p-Smad3 | Abcam | ab52903 | 1:500 |
| Caspase 8 (cleaved) | Cell signaling Technology | 9746 | 1:500 |
| Caspase 3 (Cleaved) | Cell signaling Technology | 9661 | 1:500 |
| GAPDH | Santa Cruz | sc-365062 | 1:1000 |
| β-actin | Cell signaling Technology | 3700 | 1:10000 |
| Vinculin | Sigma-Aldric | V9131 | 1:3000 |
| SMMHC (IF) | Abcam | ab125884 | 1:500 |
| αSMA-Alexa 594 (IF) | Abcam | ab202368 | 1:500 |
| Ki67 (IF) | Abcam | ab15580 | 1:500 |
| CD68 (IF) | Bio-Rad | MCA1957GA | 1:500 |
| Vimentin (IF) | Sigma-Aldric | AB1620 | 1:50 |
| CD45 | BD Biosciences | 550539 | 1:100 |
| CD31 | BD Biosciences | 553370 | 1:100 |
| COL1A1 | Cell signaling Technology | 72026 | 1:100 |
| Alexa Fluor 488 goat anti-rat IgG | Invitrogen | A11006 | 1:1000 |
| Alexa Fluor 488 goat anti-rabbit IgG | Invitrogen | A11008 | 1:1000 |
| Anti-mouse IgG-HRP | Cell signaling Technology | 7076 | 1:5000 |
| Anti-Rabbit IgG-HRP | Cell signaling Technology | 7074 | 1:5000 |
